# OCT4 activates a *Suv39h1*-repressive antisense lncRNA to couple histone H3 Lysine 9 methylation to pluripotency

**DOI:** 10.1093/nar/gkac550

**Published:** 2022-06-28

**Authors:** Laure D Bernard, Agnès Dubois, Victor Heurtier, Véronique Fischer, Inma Gonzalez, Almira Chervova, Alexandra Tachtsidi, Noa Gil, Nick Owens, Lawrence E Bates, Sandrine Vandormael-Pournin, José C R Silva, Igor Ulitsky, Michel Cohen-Tannoudji, Pablo Navarro

**Affiliations:** Institut Pasteur, Université Paris Cité, CNRS UMR3738, Epigenomics, Proliferation, and the Identity of Cells Unit, Department of Developmental and Stem Cell Biology, F-75015 Paris, France; Sorbonne Université, Collège doctoral, F-75005 Paris, France; Institut Pasteur, Université Paris Cité, CNRS UMR3738, Epigenomics, Proliferation, and the Identity of Cells Unit, Department of Developmental and Stem Cell Biology, F-75015 Paris, France; Institut Pasteur, Université Paris Cité, CNRS UMR3738, Epigenomics, Proliferation, and the Identity of Cells Unit, Department of Developmental and Stem Cell Biology, F-75015 Paris, France; Sorbonne Université, Collège doctoral, F-75005 Paris, France; Institut Pasteur, Université Paris Cité, CNRS UMR3738, Epigenomics, Proliferation, and the Identity of Cells Unit, Department of Developmental and Stem Cell Biology, F-75015 Paris, France; Institut Pasteur, Université Paris Cité, CNRS UMR3738, Epigenomics, Proliferation, and the Identity of Cells Unit, Department of Developmental and Stem Cell Biology, F-75015 Paris, France; Institut Pasteur, Université Paris Cité, CNRS UMR3738, Epigenomics, Proliferation, and the Identity of Cells Unit, Department of Developmental and Stem Cell Biology, F-75015 Paris, France; Institut Pasteur, Université Paris Cité, CNRS UMR3738, Epigenomics, Proliferation, and the Identity of Cells Unit, Department of Developmental and Stem Cell Biology, F-75015 Paris, France; Sorbonne Université, Collège doctoral, F-75005 Paris, France; Department of Immunology and Regenerative Biology and Department of Molecular Neuroscience, Weizmann Institute of Science, Rehovot, Israel; Institut Pasteur, Université Paris Cité, CNRS UMR3738, Epigenomics, Proliferation, and the Identity of Cells Unit, Department of Developmental and Stem Cell Biology, F-75015 Paris, France; MRC Human Genetics Unit, MRC Institute of Genetics and Cancer, University of Edinburgh, Edinburgh EH4 2XU, UK; Institut Pasteur, Université Paris Cité, CNRS UMR3738, Epigenomics, Proliferation, and the Identity of Cells Unit, Department of Developmental and Stem Cell Biology, F-75015 Paris, France; Guangzhou Laboratory, Guangzhou International Bio Island, Guangzhou510005, Guangdong Province, China; Department of Immunology and Regenerative Biology and Department of Molecular Neuroscience, Weizmann Institute of Science, Rehovot, Israel; Institut Pasteur, Université Paris Cité, CNRS UMR3738, Epigenomics, Proliferation, and the Identity of Cells Unit, Department of Developmental and Stem Cell Biology, F-75015 Paris, France; Institut Pasteur, Université Paris Cité, CNRS UMR3738, Epigenomics, Proliferation, and the Identity of Cells Unit, Department of Developmental and Stem Cell Biology, F-75015 Paris, France

## Abstract

Histone H3 Lysine 9 (H3K9) methylation, a characteristic mark of heterochromatin, is progressively implemented during development to contribute to cell fate restriction as differentiation proceeds. Accordingly, in undifferentiated and pluripotent mouse Embryonic Stem (ES) cells the global levels of H3K9 methylation are rather low and increase only upon differentiation. How global H3K9 methylation levels are coupled with the loss of pluripotency remains largely unknown. Here, we identify SUV39H1, a major H3K9 di- and tri-methylase, as an indirect target of the pluripotency network of Transcription Factors (TFs). We find that pluripotency TFs, principally OCT4, activate the expression of *Suv39h1as*, an antisense long non-coding RNA to *Suv39h1*. In turn, *Suv39h1as* downregulates *Suv39h1* transcription in cis via a mechanism involving the modulation of the chromatin status of the locus. The targeted deletion of the *Suv39h1as* promoter region triggers increased SUV39H1 expression and H3K9me2 and H3K9me3 levels, affecting all heterochromatic regions, particularly peri-centromeric major satellites and retrotransposons. This increase in heterochromatinization efficiency leads to accelerated and more efficient commitment into differentiation. We report, therefore, a simple genetic circuitry coupling the genetic control of pluripotency with the global efficiency of H3K9 methylation associated with a major cell fate restriction, the irreversible loss of pluripotency.

## INTRODUCTION

During development, the establishment and maintenance of distinct gene expression patterns supporting the identity of each cell type are closely linked to the regulation of chromatin states (1). Two broad states have been clearly and unambiguously identified: euchromatin, associated with transcriptionally active regions, and heterochromatin, associated with gene repression (2–5). Two major states of heterochromatin have been traditionally considered. Facultative heterochromatin refers to a repressive chromatin environment displaying high variability across developmental stages, cell types and cell states. Indeed, silent developmental genes are usually embedded in facultative heterochromatin (3,4). In contrast, ubiquitously silent elements such as retrotransposons and pericentromeric regions are locked by constitutive heterochromatin (4,5). These two types of heterochromatin have been thought to be distinguishable by several molecular signatures, with facultative heterochromatin being characterized by trimethylation of histone H3 lysine 27 (H3K27me3) and constitutive heterochromatin by H3K9me3, among other chromatin features (2–5). Nevertheless, recent data has challenged these strict definitions (3). On the one hand, constitutive heterochromatin can under some circumstances be transcribed or decorated by marks previously associated with facultative heterochromatin (6–8). On the other, while H3K27me3 and H3K9me2 were considered as major repressive mark for developmental genes, an increasing body of evidence points to H3K9me3 as an additional mean to silence developmental regulators as their expression is definitely shut down in particular lineages (9). Hence, even though the role of H3K9 methylation in genome stability is unquestionable (10), its importance in gene regulatory mechanisms during development appears to be equally important. Indeed, mouse knock-out (KO) models of H3K9 histone methyltransferases (HMTs) display penetrant phenotypes, particularly during gastrulation when pluripotency is lost and major differentiation events take place (11,12). Conversely, before reaching pluripotency during early mouse embryogenesis, the levels of H3K9 methylation are strictly controlled; promoting their increase, for instance by overexpressing the HMT SUV39H1, leads to developmental defects at the compaction stage (13,14).

While extensive research has contributed to our understanding of how the establishment and maintenance of H3K27me3 regulates developmental transitions (1–10), how the levels of H3K9 methylation are developmentally regulated is less clear. Yet, a major distinction has been identified, particularly using pluripotent cells such as mouse Embryonic Stem (ES) cells. Indeed, H3K27me3 characterizes developmental genes even before differentiation, when they are embedded in the so-called bivalent chromatin, which is simultaneously enriched for H3K27me3 and for marks of activity (15). Upon differentiation, H3K27me3 is either consolidated or erased in a cell-type-dependent manner (16). On the contrary, H3K9 methylation is more largely controlled at the level of its abundance: during differentiation the global levels of H3K9me2 and H3K9me3 increase drastically (17,18). Conversely, during the induction of pluripotency *in vitro* through reprogramming processes, H3K9 methylation has been shown to act as a major epigenetic barrier that is in part overcome by globally reducing its levels (18,19). Therefore, while H3K27 methylation is mainly controlled by altering its genomic distribution, the global levels of H3K9 methylation display correlated changes to the differentiation status. Beyond the role of H3K9 methylation to stabilise somatic cell identities (20), how its global levels are seemingly coupled to the acquisition and loss of pluripotency, and what consequences this coupling has, remain open questions.

In this study, we aimed at understanding the molecular basis of the link between H3K9 methylation and pluripotency. We find *Suv39h1* to be the only HMT tightly connected to the network of transcription factors (TFs) supporting pluripotency, particularly to its main player *Oct4*. The analysis of the mechanisms of *Suv39h1* repression by OCT4 led us to identify *Suv39h1as* (21) as a *Suv39h1*-repressive antisense long non-coding RNA (lncRNA (22)) directly activated by OCT4. Using CRISPR-Cas9 mediated deletion of the antisense promoters, we further show that its activity controls the efficiency of H3K9 methylation in ES cells and the timing of commitment into differentiation. Thus, our work identifies a simple genetic network that provides a mechanistic perspective into how the global levels of H3K9 methylation are regulated at the onset of differentiation to irreversibly exit pluripotency.

## MATERIALS AND METHODS

### Cell lines and generation of A8 and D8 *Suv39h1as* mutant cells

WT cells in this study are E14Tg2a ES cells, from which all mutant cells were derived. Dox-inducible knock-out cells as well as OCT-AID cells have been previously described (*Esrrb*: EKOiE (23), *Oct4*: ZHBTC4 (24), *Nanog*: 44iN (25), OCT-AID (26)). To generate ES cells deleted for the *Suv39h1as* promoter, a CRISPR-Cas9 approach was followed using gRNAs available in Supplementary Table S5. Additional details are available in supplementary methods and in Supplementary Figure S4.

### Regular cell culture and differentiation

Cells were cultured at 37°C, 7% CO2 on gelatine-coated plates in either FCS/LIF or in 2i/LIF, as indicated, and passaged every 2–3 days. Cells cultured in FCS/LIF were differentiated by withdrawing LIF for 3 days. N2B27 and EpiLC differentiation assays were performed with cells cultured in 2i/LIF for a minimum of 3 passages, after which LIF and 2i were withdrawn (for both N2B27 and EpiLC) and activin A, FGF and KSR supplemented (for EpiLC only). All details are available in supplementary methods.

### Commitment assays

For commitment assays, 600 cells obtained every day of differentiation in N2B27 were plated in poly-l-ornithine/laminin-coated wells of a six-well plate, cultured for 7 days in 2i/LIF and stained for alkaline phosphatase activity (supplementary methods).

### Immunostainings

To ensure direct comparisons, cell lines or conditions to be compared were individually labelled either with Rhodamine Red or with Deep Red dyes. The labelled cells were then collected, mixed at a 1:1 ratio and seeded onto poly-l-ornithine/laminin coated μ-slides. After 6 h of culture at 37°C and 7% CO_2_, they were prepared for immunostaining and imaged. Additional details are available in supplementary methods.

### Single molecule FISH and DNA-FISH

Single-strand probes for *Suv39h1* (47 oligos, 30 in exons, see sequences Supplementary Table S5) and *Suv39h1as* (35 exonic oligos, see sequences Supplementary Table S5) were used for smFISH and the position of each image recorded on the microscope. Subsequently, DNA-FISH was performed with a labelled fosmid (WIBR1-2188H11—from bacpac.chori.org) at the same positions (supplementary methods).

### ChIP-seq and ATAC-seq

Both assays and the corresponding libraries were performed and generated as previously described (27), with the exception that fixed Drosophila chromatin was spiked in to be used as an internal normaliser, and sequenced for 75 cycles in a NextSeq 500 (SR for ChIP-seq and PE for ATAC-seq). For ChIP-seq, adapters with UMIs to enable distinguishing true identical reads from PCR duplicates (27) were used to improve mapping of repetitive DNA. After alignment (Bowtie2) to both mouse and Drosophila genomes, mouse peaks were identified with MACS2 for ATAC-seq and with a previously described approach (28) for H3K9me3. Read counts at these regions were normalised to the total number of reads aligning to the Drosophila genome, considering for ChIP-seq both the ChIP and corresponding input of each replicate for H3K9me3. For ChIP-seq, for reads mapping at multiple positions only one was randomly kept; for ATAC-seq, all multi-mappers were excluded. Global analysis of repetitive elements was performed with RepEnrich (29) followed by DESeq2 (30).

## RESULTS

### Suv39h1 expression is under the control of OCT4 in ES cells

**Figure 1.**
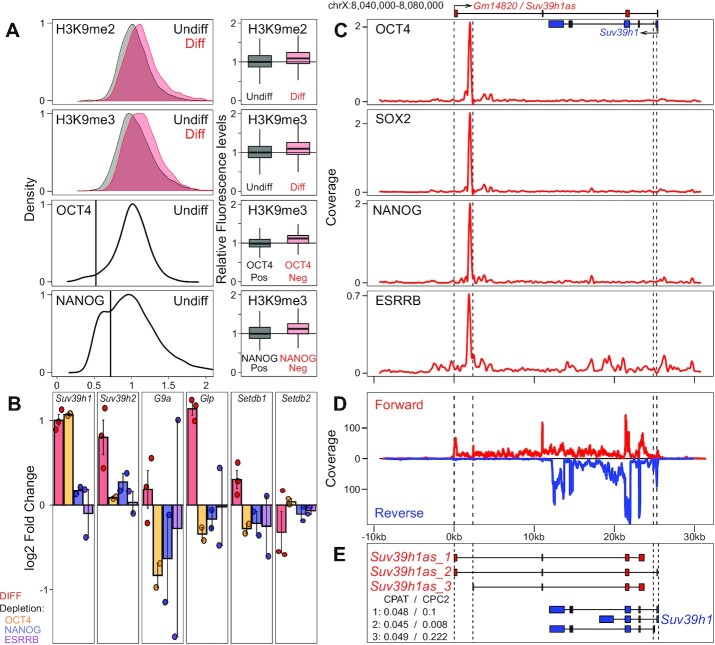
*Suv39h1* is downregulated by OCT4, which binds to the promoter of a *Suv39h1* antisense lncRNA, *Suv39h1as*. (**A**) On the left, distribution of H3K9me2, H3K9me3, OCT4 and NANOG in undifferentiated (black; *n* = 4503, 3150, 3276 and 5615 cells, respectively) and differentiating (3d of LIF withdrawal – red; *n* = 6231, 3755, respectively) ES cell populations assessed by immunofluorescence. On the right, boxplot corresponding to different populations shown on the left: for H3K9me2/me3 distributions, the boxplots compare undifferentiated and differentiating cells (KS test *P* < 10^–15^); for OCT4 and NANOG distributions, the boxplots compare H3K9me3 between positive/negative subpopulations of OCT4 or NANOG (vertical line on the left panels; KS test *P* < 10^–15^ for both TFs). (**B**) Log_2_ fold change of the indicated gene after differentiating ES cells as in (A) or 24h after inducing the depletion of individual TFs, as indicated, using Dox-inducible knock-out cells. Each dot represents an independent replicate and the bar and error bars the corresponding means and standard errors. *Suv39h1* upregulation upon differentiation or OCT4 depletion (*P* < 0.001) and *Suv39h2* and *Glp* upregulation upon differentiation (*P* < 0.05) are statistically significant (t test). (**C**) Average binding profile of OCT4, SOX2, NANOG and ESRRB (reads per million) across the *Suv39h1*/*Gm14820* locus (mm10, chrX:8 040 000–8 080 000). *Suv39h1* and *Gm14820/Suv39h1as* are schematically represented on top. (**D**) RNA-seq profile across the *Suv39h1*/*Suv39h1as* locus, with forward and reverse fragment counts expressed with positive and negative values. (**E**) Schematic representation of *Suv39h1as* (red) and *Suv39h1* (blue) isoforms as determined in Supplementary Figure S2. The coding probabilities calculated with CPAT and CPC2 algorithms are shown for the three isoforms of *Suv39h1as*. The vertical dashed lines in (C), (D) and (E) mark the position of *Suv39h1as* or *Suv39h1* promoters.

Using immunofluorescence, we first confirmed that differentiation of ES cells by LIF withdrawal leads to an increase of both H3K9me2 and H3K9me3, an increase that can also be observed in spontaneously differentiating cells in regular ES cell cultures, which express low levels of the pluripotency TFs OCT4 or NANOG (Figure 1A and Supplementary Figure S1A, B). Therefore, we hypothesized that one or several histone methyl-transferases or lysine demethylases (HMTs and KDMs, respectively) (2,5) could be directly controlled by pluripotency TFs and differentially expressed upon differentiation, linking the loss of pluripotency to increased H3K9 methylation. To assess this, we monitored mRNA levels of HMTs and KDMs using published RNA-seq datasets of undifferentiated and differentiating ES cells (31) (Figure 1B, Supplementary Figure S1C, Supplementary Table S1). We found three HMTs to be upregulated upon differentiation: *Suv39h1*, *Suv39h2* and *Glp*. In contrast, all tested KDM displayed minor changes below 2-fold (Supplementary Figure S1C). We reasoned that the increase of *Suv39h1*, *Suv39h2* and *Glp* expression could either be due to a direct control of their transcription by pluripotency TFs or to alternative, indirect, mechanisms. To address this, we assessed the impact of the loss of individual pluripotency TFs (*Oct4*, *Nanog* and *Esrrb*) using dox-inducible knock-outs (23–26) (Supplementary Figure S1D). For NANOG, we used available datasets (31); for OCT4 and ESRRB they were generated for this study (Supplementary Table S1). Only one HMT, *Suv39h1*, was found upregulated 24h after inducing the loss of pluripotency TFs, particularly of OCT4, which depletion leads to a 2-fold increase in *Suv39h1* mRNA levels (Figure 1B). Moreover, analysis of datasets from gastrulating mouse embryos (32) also showed *Suv39h1* to be the only HMT to be upregulated upon differentiation of the three main germ layers (Supplementary Figure S1E). Hence, after confirming *Suv39h1* expression changes by RT-qPCR (Supplementary Figure S1F), we hypothesized that OCT4 may act as a repressor of *Suv39h1* expression to maintain low levels of H3K9 methylation until the onset of differentiation. Exploration of available ChIP-seq datasets (27) (Figure 1C) and direct validation by ChIP-qPCR (Supplementary Figure S1G) identified a hotspot of pluripotency TFs, including OCT4, in the vicinity of *Suv39h1*. However, this TF binding hotspot was found located 3′ to *Suv39h1*, at around 27 kb of its promoter region. Notably, we noticed that this region coincides with the promoter region of an uncharacterized gene, *Gm14820* (*AK010638*), antisense to and largely overlapping *Suv39h1* (top of Figure 1C). This antisense transcript, *Suv39h1as*, has been previously identified in oocytes and suggested to oppose to *Suv39h1* expression at the oocyte to zygote transition (21).

### Suv39h1as is an antisense long non-coding RNA

**Figure 2.**
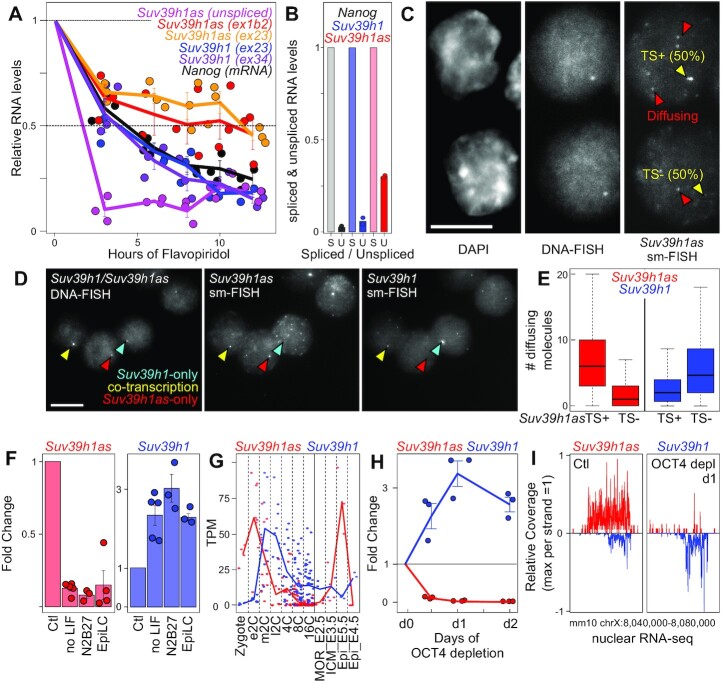
*Suv39h1as* is a nuclear, stable and lowly expressed lncRNA with anticorrelated expression dynamics to *Suv39h1*. (**A**) RT-qPCR analysis of the half-life of several RNA species during a transcription inhibition assay with Flavopiridol: *Suv39h1* mRNA, using two trans-exonic primer pairs between exons 2 and 3 (blue – ex23) or exons 3 and 4 (purple – ex34); *Suv39h1as*, using two trans-exonic primer pairs between exons 1b and 2 (red – ex1b2) or exons 2 and 3 (orange – ex23) or primer pairs amplifying the unspliced RNA (magenta); *Nanog* mRNA (black). Each dot represents an independent replicate and the line the corresponding mean and standard error. Ribosomal RNA (28s) was used for normalization. (**B**) Histogram representing unspliced RNA levels relative to corresponding spliced RNAs for *Nanog*, *Suv39h1* and *Suv39h1as*, as measured by RNA-seq. Each dot represents an independent replicate and the bar the corresponding mean. (**C**) Representative sm-FISH followed by DNA-FISH visualizing *Suv39h1as* RNA molecules and the *Suv39h1*/*Suv39h1as* locus, respectively, in undifferentiated WT cells. Red arrowheads indicate RNAs diffusing away from the locus, which is indicated by a yellow arrow. The proportion of actively transcribing cells is indicated (*n* = 358 cells). (**D**) Representative sm-FISH of *Suv39h1as* and *Suv39h1* RNA molecules, followed by DNA-FISH visualising the *Suv39h1*/*Suv39h1as* locus in WT cells (*n* = 358). Selected loci transcribing either *Suv39h1*, *Suv39h1as* or both genes are indicated with arrow heads: blue, *Suv39h1*-only (30%); red, *Suv39h1as*-only (30%); yellow for cells transcribing both (20%). (**E**) Boxplots (median; 25–75% percentiles; error bars) showing the number of *Suv39h1* diffusible molecules counted in cells presenting an active (TS+) or inactive (TS-) *Suv39h1as* gene (*n* = 358 WT cells). The increased of *Suv39h1* diffusing molecules in *Suv39h1*_TS- versus *Suv39h1as*_TS + was assessed with KS test (*P* < 10^–11^). (**F**) Fold change expression of *Suv39h1as* (red) or *Suv39h1* (blue) measured by RT-qPCR in differentiating WT cells versus undifferentiated controls (ctl). Differentiation was triggered for three days with three independent protocols: LIF withdrawal from FCS/LIF cultures (no LIF), 2i and LIF withdrawal from 2i/LIF cultures (N2B27) or EpiLC differentiation from 2i/LIF cultures (EpiLC). Values were normalized to *Tbp* and fold changes calculated to their respective control cultures. Each dot represents an independent replicate and the bar and error bars the corresponding means and standard errors. Gene expression differences were assessed against the respective undifferentiated controls (t test *P* < 0.05 for *Suv39h1* and *P* < 0.01 for *Suv39h1as* for all differentiation assays). (**G**) Mean expression dynamics of *Suv39h1* and *Suv39h1as* in published RNA-seq datasets of early mouse embryogenesis (left part, Zygote to 16-cell stage (35); right part, Morula, ICM and Epiblast (36)). (**H**) RT-qPCR analysis of *Suv39h1as* (red) and *Suv39h1* (blue) expression upon OCT4 depletion in ZHBTC4 cells treated with Dox for the indicated time. Values were normalized to *Tbp*. Each dot represents an independent replicate and the line the corresponding mean with standard errors. Each time-point was compared to untreated cells with a *t* test (*P* < 0.001 for *Suv39h1as* and *P* < 0.05 for *Suv39h1*). (**I**) Analysis of publicly available (37) nuclear RNA-seq over the *Suv39h1*/*Suv39h1as* locus in untreated and OCT4-depleted ZHBTC4 cells (1 day of Dox treatment), presented as in Figure 1D.

Stranded, total RNA-seq confirmed *Suv39h1as* to be expressed in ES cells, at levels comparable to *Suv39h1* (Figure 1D). Using de novo transcript assembly with all the RNA-seq datasets presented in Supplementary Table S1, together with direct cDNA cloning, sequencing and RT-qPCR, we identified several isoforms expressed in ES cells (Figure 1E and Supplementary Figure S2A–C). All isoforms initiate from two distinct promoters, located in proximity to the region bound by pluripotency TFs, exhibit overlapping exons with *Suv39h1* and terminate within *Suv39h1* or in the vicinity of its 5′ end. Notably, *Suv39h1as* is annotated as a long non-coding RNA (lncRNA). Accordingly, using two different algorithms (CPAT (33) and CPC2 (34)), the nearly absent coding potential of all *Suv39h1as* isoforms was confirmed (Figure 1E). To further characterize *Suv39h1as*, we assessed the stability of its RNA products and found the half-life of its spliced and unspliced forms to be around 12h and 1h30, respectively (Figure 2A). However, *Suv39h1as* splicing is relatively inefficient compared to *Suv39h1* or another protein coding gene, *Nanog* (Figure 2B), as is generally the case for lncRNA (22). Moreover, *Suv39h1as* was efficiently captured in poly-A selected RNA-seq, suggesting it is normally poly-adenylated (Supplementary Table S1). Next, we aimed at visualizing *Suv39h1as* RNA molecules in single cells. For this, we designed oligonucleotides targeting *Suv39h1as* exons and performed strand-specific single molecule RNA-FISH (smFISH) coupled to DNA-FISH to identify the *Suv39h1as*/*Suv39h1* locus, using a fosmid covering the whole region (Figure 2C). We observed that *Suv39h1as* is mainly detected as a bright point in the nucleus, likely representing actively transcribed loci as it coincides with the DNA-FISH signal. A small number of single *Suv39h1as* RNA molecules could also be detected diffusing in the nucleus and, more rarely, in the cytoplasm. Quantification of the smFISH/DNA-FISH suggested a transcriptional frequency of around 50% in the population, with a median of six freely diffusing RNAs in cells presenting a transcriptionally active locus (Figure 2E). Hence, we conclude that the pluripotency TFs bind close to the two promoters of a *Suv39h1* antisense lncRNA, which is mostly localised at its site of transcription, poly-adenylated and poorly spliced even though the spliced isoforms are relatively stable. *Suv39h1as* is, moreover, conserved in humans (Supplementary Figure S2D).

### Anticorrelated expression patterns of *Suv39h1* and *Suv39h1as*

To investigate *Suv39h1as* and*Suv39h1* expression patterns at the single cell level, we designed oligonucleotides across *Suv39h1* exons and introns to monitor *Suv39h1*/*Suv39h1as* expression by smFISH in parallel to DNA-FISH (Figure 2D). We found around 20% of cells actively transcribing both sense/antisense genes and around 30% transcribing either one or the other. Moreover, cells actively transcribing *Suv39h1as* displayed significantly fewer *Suv39h1* mRNA molecules (Figure 2E). Next, we differentiated ES cells using three independent protocols based on LIF withdrawal, N2B27 or EpiLC-directed differentiation. These three assays showed a strong reduction of *Suv39h1as* expression after 3 days of differentiation, when *Suv39h1* expression increases (Figure 2F). Moreover, exploration of published RNA-seq during early embryogenesis (35,36) confirmed the anticorrelated expression patterns of *Suv39h1*/*Suv39h1as* during key events (Figure 2G): first, following fertilization (as previously suggested (21)), when *Suv39h1as* is highly expressed but decreases rapidly followed by *Suv39h1* upregulation; second in the pluripotent ICM when *Suv39h1as* peaks at high levels, coinciding with a transient downregulation of *Suv39h1*. Hence, both *ex vivo* and *in vivo*, we observe anti-correlated expression dynamics of *Suv39h1*/*Suv39h1as*. Finally, to address whether *Suv39h1as* responds to OCT4 levels, we measured both RNA levels upon OCT4 depletion. We observed that the depletion of OCT4 leads to downregulation of *Suv39h1as*, reaching minimal levels of expression within 12 h and accompanied by a marked increase of *Suv39h1* expression that reached maximal levels after 24 h (Figure 2H). Analysis of published nuclear RNA-seq (37) further confirmed this observation, underscoring a dramatic transcriptional silencing of *Suv39h1as* upon OCT4 depletion and a strong transcriptional induction of *Suv39h1* (Figure 2I). Overall, *Suv39h1* and *Suv39h1as* display anticorrelated transcription levels upon differentiation and during early embryogenesis. This anticorrelation stems from single cell dynamics where the transcription of the antisense is accompanied by a reduction of the transcriptional frequency of *Suv39h1*. Since *Suv39h1as* is downregulated upon differentiation and upon the loss of OCT4, our data suggests that pluripotent TFs activate *Suv39h1as* transcription which, in turn, downregulates *Suv39h1* expression.

### The *Oct4-Suv39h1as-Suv39h1* circuitry

**Figure 3.**
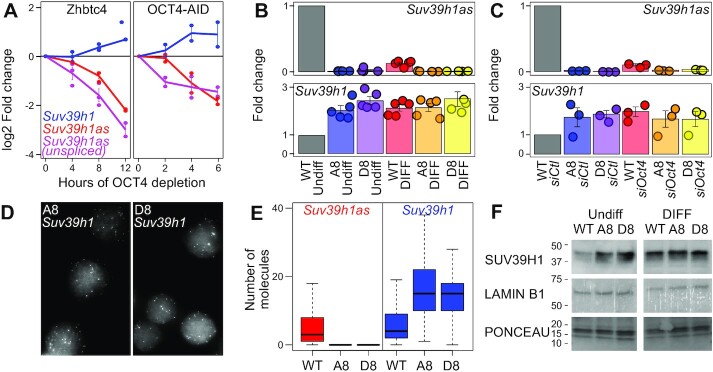
OCT4 represses *Suv39h1* via *Suv39h1as*. (**A**) Log_2_ expression fold change of *Suv39h1* (blue), *Suv39h1as* (red) and unspliced *Suv39h1as* (magenta) upon OCT4 depletion in ZHBTC4 (left) and OCT4-AID (right) cells. The X-axis shows the number of hours of treatment with Dox (ZHBTC4) and IAA (OCT4-AID). Two biological replicates per cell line and time-point are shown. The global effect of the treatments was assessed by comparing all time points together against untreated cells with a t test (*P* < 0.02 for both ZHBTC4 and OCT4-AID). (**B**) Expression fold change of *Suv39h1as* (top) and *Suv39h1* (bottom) in WT and *Suv39h1as*-mutant cells (A8 and D8) cultured in undifferentiated or differentiating conditions (3 days without LIF). Values were normalized to *Tbp*. Each dot represents an independent replicate and the bar and error bars the corresponding means and standard errors. The increase of *Suv39h1* in each clone versus WT cells was compared with a t test (p = 0.011 and 0.0016 for A8 and D8, respectively). (**C**) Expression fold change of *Suv39h1as* (top) and *Suv39h1* (bottom) in WT and *Suv39h1as*-mutant cells (A8 and D8) knocked-down with either control or *Oct4*-targeted siRNAs. Values were normalized to *Tbp*. Each dot represents an independent replicate and the bar and error bars the corresponding means and standard errors. The increase of *Suv39h1* expression upon OCT4 knock-down was assessed against control siRNAs with a *t* test (*P* = 0.027 in WT cells). (**D**) Representative sm-FISH images of *Suv39h1* in A8 and D8 cells (WT cells presented in Figure 2D). Differences in the frequency of active transcription sites were assessed with a Chi2 test (*P* < 10^–10^ for both clones against WT cells). (**E**) Boxplots (median; 25–75% percentiles; error bars) showing the number of *Suv39h1as* (red) or *Suv39h1* (blue) diffusible molecules counted in WT (*n* = 358) or mutant cells (A8, *n* = 289; D8, *n* = 270). Differences in the number of *Suv39h1* molecules were assessed with a Mann-Whitney test (*P* < 10^–15^ for both clones against WT cells). (**F**) Representative Western-Blot of SUV39H1, LAMIN B1 and corresponding Ponceau for WT and mutant cells (A8 and D8) in undifferentiated and differentiating (3 days without LIF) conditions. On the left of each image is indicated the protein scale in kDa.

OCT4 depletion leads to *Suv39h1as* silencing within 12h (Figure 2H). However, to establish that this response is a primary effect mediated by OCT4 we aimed at analysing the effect of shorter OCT4 depletion. Using the same dox-inducible cells, we initially observed that *Suv39h1as* transcription assessed by unspliced RNA quantification starts to decrease as soon as 4 h after inducing OCT4 loss (Figure 3A, left). This prompted us to use a faster degradation system of OCT4, generated by fusion with an IAA-inducible degron (26). We observed a fast response of *Suv39h1as*, displaying a marked reduction of transcription as early as 2h after inducing OCT4 depletion (Figure 3A, right). In both systems, we observed that the loss of *Suv39h1as* transcription was followed by a nearly concomitant reduction of *Suv39h1as* mature RNA and an increase of *Suv39h1* mRNA. Hence, these analyses establish that *Suv39h1as* transcription responds rapidly to the loss of OCT4. This strengthens the notion that *Suv39h1as* is a direct OCT4 target that downregulates *Suv39h1* expression. To functionally establish the relationships between OCT4, *Suv39h1as*, *Suv39h1* and H3K9 methylation, we designed two gRNAs to delete 5.5 kb encompassing the two promoters of *Suv39h1as*. Two independent KO clones, A8 and D8, were generated (Supplementary Figure S3). RT-qPCR showed a complete extinction of *Suv39h1as* expression (Figure 3B). We then addressed the impact of *Suv39h1as* depletion on *Suv39h1* expression, both before and during differentiation. In the two mutant clones we observed an increase of *Suv39h1* expression in undifferentiated cells, reaching the levels observed upon differentiation in wild-type (WT) cells (Figure 3B). In differentiating cells, when OCT4 binding at the *Suv39h1as* promoter is abrogated (Supplementary Figure S4A), and *Suv39h1as* naturally silenced, the deletion had no impact (Figure 3B), as expected. Analysis of several differentiation markers (Supplementary Figure S4B, left) ruled out the possibility that differences in *Suv39h1* expression derive from indirect consequences linked to the differentiation status of the cells, as already suggested by the fast response of *Suv39h1as* transcription to OCT4 depletion (Figure 3A). Moreover, these results were independent of the differentiation protocol (Supplementary Figure S4C). Therefore, these results indicate that *Suv39h1as* acts as a pluripotency-associated repressor of *Suv39h1* expression. To more directly address whether OCT4 represses *Suv39h1* expression via *Suv39h1as*, we used siRNAs targeting *Oct4* to test whether in the absence of *Suv39h1as*, the loss of OCT4 would lead to any modification of *Suv39h1* expression. Whereas in WT cells the knock-down of *Oct4* (above 80% efficiency, Supplementary Figure S4D) led to higher *Suv39h1* expression, in mutant cells it was fully inconsequential (Figure 3C). Other OCT4-responsive genes, such as *Cdx2* (24), displayed similar changes in WT and mutant cells upon *Oct4* knock-down (Supplementary Figure S4B, middle panel). Thus, OCT4-dependent repression of *Suv39h1* only occurs in the presence of the promoter region of *Suv39h1as*, suggesting that it requires *Suv39h1as* transcription. Next, we performed smFISH to study *Suv39h1* upregulation with single cell resolution (Figure 3D). We observed a marked increase in the transcriptional frequency of *Suv39h1*, rising from 47.6% in WT to 76.3% and 74.1% in A8 and D8, respectively. Moreover, the number of *Suv39h1* mRNAs per cell also increased substantially, with virtually no cell displaying an absence of *Suv39h1* mRNAs (Figure 3E). This increase in *Suv39h1* transcription in mutant cells was accompanied by higher levels of SUV39H1 protein levels, reaching those observed in WT differentiating cells (Figure 3F). Altogether, our results indicate that OCT4 directly activates *Suv39h1as* transcription, which in turn transcriptionally downregulates *Suv39h1*, leading to reduced mRNA and protein levels. This simple genetic circuitry ensures increased SUV39H1 expression during differentiation.

### *Suv39h1as* modifies the chromatin of the *Suv39h1as*/*Suv39h1* locus

**Figure 4.**
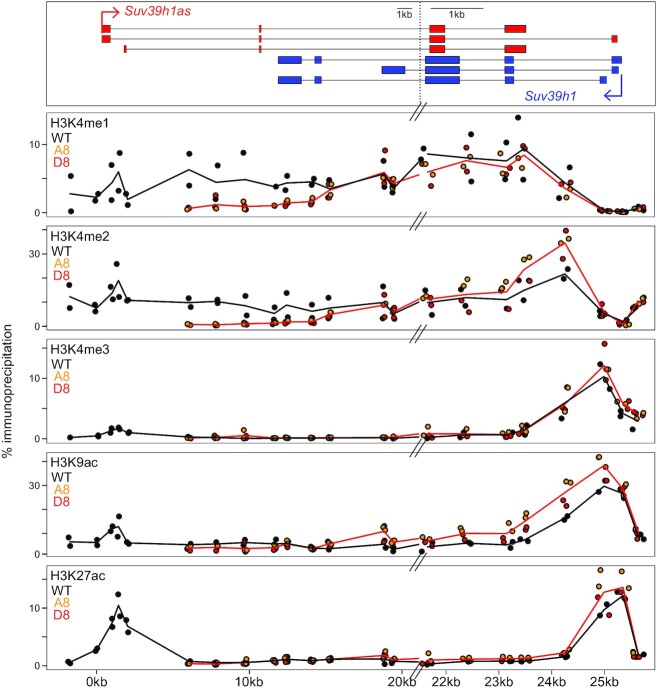
*Suv39h1as* triggers complex chromatin changes across the locus. Chromatin immunoprecipitation profile of H3K4me1, H3K4me2, H3K4me3, H3K9ac and H3K27ac, as indicated, across *Suv39h1*/*Suv39h1as* locus in WT (black) and *Suv39h1as*-mutant cells (A8, yellow dots; D8, red dots; the red line represents the average of all data points for mutant clones). The X-axis represents genomic distances in kb with respect to the *Suv39h1as* transcription start site, as schematized on top. Note a break on the scale of the genomic coordinates at around X = 21kb. For the analysis of the locus-wide effects of the loss of *Suv39h1as* on H3K4me1 and me2, all values obtained with primer pairs located between coordinates +6 and +15 were considered (*t* test *P* < 0.001); for the effects measured at the promoter region, the position showing the highest difference for each histone mark was used (*t* test *P* < 0.05 for H3K4me2/me3, H3K9ac and H3K27ac).

We have observed that in mutant ES cells lacking *Suv39h1as* expression, the transcriptional frequency of *Suv39h1* increases from 50 to 75% (Figure 3D). Similarly, upon OCT4 depletion *Suv39h1* pre-mRNA increases substantially (Figure 2I) and rapidly (Figure 3A). Moreover, the absence of *Suv39h1as* is not accompanied by increased stability of *Suv39h1* mRNAs (Supplementary Figure S5A). In contrast, in *Suv39h1as* mutant cells we observed increased chromatin accessibility of the *Suv39h1* promoter region (Supplementary Figure S5B and Supplementary Table S2). Therefore, *Suv39h1as* is likely to act as a transcriptional repressor of *Suv39h1*. To explore this, and given that other antisense transcription units have been shown to modify the chromatin of their corresponding sense gene (38–40), we used a ChIP approach to establish the histone modification profile of the locus (Figure 4). First, we monitored H3K4 methylation profiles. We found H3K4me1/me2, which usually mark transcriptionally competent regions (41), to globally decorate the locus with minimal focal accumulation at promoters. Conversely, H3K4me3, a mark of activity (41), was focally enriched at the *Suv39h1* promoter and displayed low levels over the antisense promoter. We then profiled the active histone acetylation marks H3K9ac and H3K27ac. Similarly to H3K4me3, we found H3K9ac to preferentially mark the *Suv39h1* promoter. In contrast, both sense and antisense gene promoters where enriched for H3K27ac. In mutant cells, we observed a global decrease of H3K4me1/me2 over the region transcribed by *Suv39h1as*, particularly before it reaches the *Suv39h1* gene body (Figure 4), indicating its transcription promotes the establishment of these marks. The lack of H3K4me1/me2 reduction within the region transcribed by both genes suggests that the increased transcription of *Suv39h1* may have a compensatory role. Moreover, H3K4me2, H3K9ac and H3K27ac, all marks of gene activity, showed a slight but statistically significant increase at the *Suv39h1* promoter in the absence of *Suv39h1as* (Figure 4). Altogether, this analysis suggests that the loss of *Suv39h1as* leads to increased *Suv39h1* transcription at least in part mediated by increased euchromatinisation and accessibility of the *Suv39h1* promoter. While undeniably small, the effects of the loss of *Suv39h1as* on the *Suv39h1* promoter can be reproduced with different assays measuring chromatin activity.

### Global increase of H3K9me2 and H3K9me3 in *Suv39h1as* mutant ES cells

**Figure 5.**
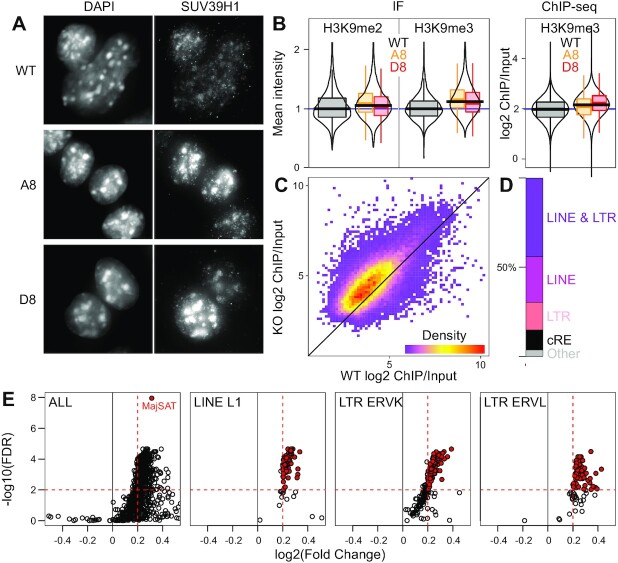
Global increase of H3K9 methylation in *Suv39h1as* mutant cells. (**A**) Representative SUV39H1 immunofluorescence of WT and *Suv39h1*-mutant ES cells (A8 and D8). (**B**) Violin and boxplots (median; 25–75% percentiles; error bars) of H3K9 methylation levels measured by immunofluorescence (left) or ChIP-seq (right). Immunofluorescence data shows relative mean intensity values of WT (black; *n* = 12881 for H3K9me2 and *n* = 12053 for H3K9me3) or mutant cells (A8, yellow, *n* = 3553 cells for H3K9me2 and 2081 for H3K9me3; D8, red, *n* = 5050 cells for H3K9me2 and 2641 for H3K9me3). KS tests were performed to compare WT and mutant cells (*P* < 10^–8^ for both marks and cell clones). ChIP-seq data shows log2 fold change of H3K9me3 over input, calculated after normalising both values to internal Drosphila spike in controls, for all the regions identified as H3K9me3-enriched in either WT or mutant clones. Differences between WT and mutant cells were evaluated with KS tests (*P* < 10^–15^ for both clones). (**C**) Scatter plot (X-axis: H3K9me3 levels in WT cells; Y:axis: mean H3K9me3 levels of the two mutant clones) corresponding to the data shown in (B). (**D**) Proportion of H3K9me3-enriched regions overlapping with both LINE and LTR retrotransposons or with only one of the two families or with cis-regulatory elements (55) (cRE) or with none of the above (Other). (**E**) Global analysis of repetitive elements (29), visualised as a volcano plot (X-axis: log_2_ fold change between mean H3K9me3 levels in mutant clones and WT cells; Y-axis: -log10(FDR); both calculated with DESeq2 (30) and considering Drosophila spike in controls. The first panel shows all repeat families, with major Satellites highlighted in red. The three following panels display selected families, as indicated, with red points highlighting elements with FDR < 0.01 and log2(FC) >0.2.

The absence of *Suv39h1as* transcription in ES cells leads to increased SUV39H1 protein levels (Figure 3F), as confirmed by immuno-fluorescence: heterochromatic regions such as chromocenters prominently accumulate SUV39H1 in mutant cells (Figure 5A, Supplementary Figure S6A). Consequently, in both mutant clones we observed higher levels of H3K9me2 and H3K9me3 (Figure 5B, left panel), similar to those observed in WT differentiating cells (Supplementary Figure S6B), establishing a direct link between *Suv39h1as* and the global levels of H3K9 methylation in ES cells. According to the preferential enrichment of SUV39H1 at chromocenters, where H3K9me3-enriched peri-centromeric heterochromatin clusters (5), the increase of H3K9me3 in *Suv39h1as* mutant cells was higher than that of H3K9me2 (Figure 5B left panel). Hence, we aimed at more precisely characterise H3K9me3 in WT and *Suv39h1as* mutant cells by ChIP-seq. We identified 48,584 regions with more than 3-fold enrichment of H3K9me3 compared to the corresponding input in either WT or in at least one mutant clone (Supplementary Table S3). Comparative analysis of WT and mutant cells showed that the vast majority of these regions display higher H3K9me3 enrichment in the absence of *Suv39h1as* (Figure 5C), in magnitudes similar to those observed by immuno-fluorescence (Figure 5B, right panel). We also observed that more than 80% of H3K9me3-enriched regions overlap with retrotransposons (either LTRs, LINEs, or both; Figure 5D) and only minimally with cis regulatory elements (cRE in Figure 5D), as previously showed (7). Next, we quantified the effect of *Suv39h1as* KO at repetitive elements. We observed a strong bias towards a global and moderate increase of H3K9me3 over a multitude of families (Figure 5E, Supplementary Table S4), most notably at major satellites of peri-centromeric regions (red dot in the left panel of Figure 5E and Supplementary Figure S6C), but also at retrotransposons such as LINE L1s or LTRs (ERVKs and ERVLs; Figure 5E and Supplementary Figure S6D). We conclude that *Suv39h1as* mutant cells display increased efficiency to trigger heterochromatin in ES cells.

### The lack of *Suv39h1as* leads to accelerated differentiation commitment

**Figure 6.**
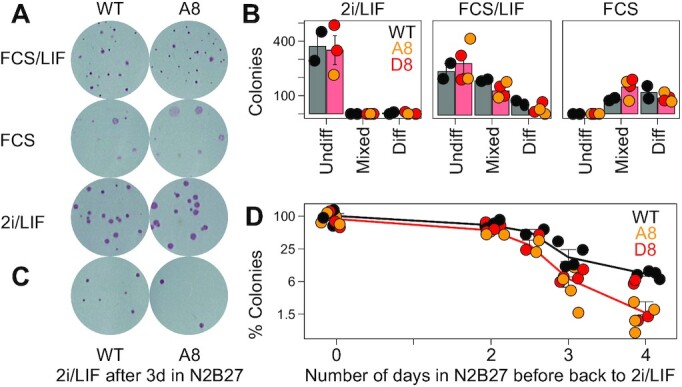
Accelerated commitment into differentiation in the absence of *Suv39h1as*. (**A**) Representative alkaline-phosphatase staining of ES cell colonies cultured as indicated. (**B**) Number of WT or *Suv39h1as*-mutant (A8, orange points; D8, red points) colonies characterized as undifferentiated, mixed or differentiated after culturing them as indicated. Each dot represents an independent replicate and the bar and error bars the corresponding means (black for WT and red for the mean of all data points for mutant clones) and standard errors. (**C**) Alkaline-phosphatase staining of ES cell colonies cultured in 2i/LIF after 3 days in N2B27 for WT and *Suv39h1as*-mutant cells (A8). (**D**) Percentage of alkaline-phosphatase positive colonies cultured in 2i/LIF after differentiating them for the indicated number of days (X-axis) in N2B27. D0, undifferentiated cells were set as 100%. Each dot represents an independent replicate (WT, black; *Suv39h1as*-mutant clones in orange, A8, and red, D8) and the line the corresponding mean and standard error (all mutant data points were averaged to obtain the red line). Differences between WT and the mean of the two mutant clones were evaluated with Mann-Whitney tests using raw colony numbers (*P* = 0.0666, 0.0020 and 0.0041 for days 2.5, 3 and 4, respectively).

We finally wondered whether the increase of H3K9 methylation taking place in *Suv39h1as* mutant cells had any physiological impact. First, we used clonal assays to assess self-renewal and differentiation efficiency (Figure 6A, B). Either in conditions of reinforced self-renewal (2i/LIF), in traditional serum-containing culture medium (FCS/LIF) or in the absence of LIF (FCS), the number of alkaline-phosphatase colonies, a marker of pluripotent cells, was similar between WT and mutant clones. Hence, the presence of increased H3K9 methylation is largely inconsequential for self-renewal and for the loss of pluripotency. In agreement, both WT and mutant cells proliferate and differentiate normally, as evaluated morphologically (Supplementary Figure S7A) and by marker expression (Supplementary Figure S7B and Supplementary Figure S4B). However, during differentiation, the role of H3K9 methylation is to restrict cell fate and developmental competence (9,18,28,42), more than to elicit differentiation, with SUV39H1 playing a preponderant role in lineage-dependent maintenance of gene silencing in somatic cells (43). Therefore, we reasoned that the loss of *Suv39h1as* could modulate the timing of commitment into differentiation. To test this, we used an established assay (44) whereby WT and mutant clones were differentiated in parallel and, every day, the cells were harvested and reseeded clonally in 2i/LIF: only those cells that were not yet committed into irreversible differentiation can self-renew and form undifferentiated colonies (Figure 6C, D). As previously shown (44), we observed that commitment took place between days 2 and 3 in WT cells, with a reduction in clonogenicity of nearly 90% (Figure 6D). In mutant cells, however, the reduction in the number of undifferentiated colonies was more marked from day 2.5 onwards (Figure 6D). Therefore, the premature establishment of higher levels of H3K9me2/me3 in ES cells facilitates the irreversible commitment into differentiation, in line with the role of these repressive marks in locking cell fate changes (20).

## DISCUSSION

In this study, we have identified a genetic network linking the control of the global levels of H3K9 methylation to pluripotency. The pluripotency network, mainly through OCT4, activates *Suv39h1as*, an antisense lncRNA to the *Suv39h1* gene; in turn, *Suv39h1as* represses *Suv39h1* expression. Consequently, the level of H3K9 methylation is reduced. Upon differentiation, the collapse of the pluripotency network leads to the silencing of *Suv39h1as*, enabling increased SUV39H1 expression and H3K9 methylation, which affects the timing and efficiency of the irreversible commitment into differentiation. Given the lack of strong effects at a small number of defined regulators of differentiation in *Suv39h1as* mutant cells, our data suggest that their faster commitment into differentiation is achieved by globally ameliorating the efficiency of H3K9 methylation, as illustrated by higher levels of H3K9me3 at peri-centromeric major satellites and at retrotransposons. During exit from pluripotency, when proper epigenetic silencing is implemented (45), the enhancement of heterochromatinization may enable the long-term changes of gene expression required to acquire new cell identities. Moreover, it is noteworthy that the *Oct4*-*Suv39h1as*-*Suv39h1* genetic axis may also act as a time-delay generator, enabling ES cells to filter out transient and short fluctuations of the pluripotency network (46): only a long decrease in activity of pluripotency TFs, such as that occurring during the exit from pluripotency, may be sufficient to elicit the increase in SUV39H1 expression that will follow the extinction of *Suv39h1as*.

Antisense lncRNAs are frequent in mammals, with 29% of canonical protein coding genes displaying antisense transcription (47). Given their antisense orientation and the resulting complementarity, antisense lncRNAs can theoretically regulate their cis-linked sense gene through a wide variety of mechanisms. By deleting the *Suv39h1as* promoter region, we found that *Suv39h1as* controls the transcriptional frequency of *Suv39h1*, whose promoter becomes more accessible and enriched in euchromatin marks. Additionally, *Suv39h1as* mutants display no changes of *Suv39h1* mRNA stability. Moreover, OCT4 depletion leads to a fast silencing of *Suv39h1as*, accompanied by a transcriptional induction of *Suv39h1* that takes place almost concomitantly to the decrease of mature *Suv39h1as* RNA. Altogether, this data supports the notion of a transcriptional control of the *Suv39h1* promoter by *Suv39h1as*, either by the act of *Suv39h1as* transcription itself or through mechanisms involving the unspliced *Suv39h1as* RNA. Notably, this ambiguity characterises other well-known pairs of sense/antisense genes, such as *Xist*/*Tsix* (38–40). Like the loss of *Tsix*, that of *Suv39h1as* results in complex chromatin changes, which suggest that *Suv39h1as* triggers H3K4me1 and me2 throughout the locus and, at the same time, reduces the enrichment for active histone marks and the accessibility of the *Suv39h1* promoter. Even though the reminiscence of these effects to those triggered by *Tsix* are striking, possibly revealing a general property of antisense transcription, we cannot exclude that they are indirect consequences of a transcriptional induction of *Suv39h1* mediated by other mechanisms. For instance, a direct competition between *Suv39h1*/*Suv39h1as* and other gene promoters for shared enhancers could play an important role (48). In agreement, exploration of the topology of the extended *Suv39h1*/*Suv39h1as* locus suggests that the 3D organization is compatible with the involvement of 3D events (Supplementary Figure S8A); nevertheless, neither chromatin accessibility (Supplementary Figure S8B,C), nor expression analyses of genes previously identified (49) as sharing at least a common enhancer with *Suv39h1*/*Suv39h1as* (Supplementary Figure S8D), display any change in our mutant cells. This indicates that *Suv39h1as* has a very local and specific role within the locus: to control the promoter activity of *Suv39h1*. Nevertheless, other additional mechanisms cannot be excluded, such as direct collisions of the sense/antisense polymerases (50) or post-transcriptional mechanisms involving splicing (51).

The deletion of *Suv39h1as* promoter and the ensuing increase in H3K9 methylation appears to be largely tolerated by ES cells: they self-renew and differentiate efficiently. This observation is in line with others, where histone modifiers have been either invalidated or ectopically expressed in ES cells with minor consequences for self-renewal (52,53). However, despite the fact that *Suv39h1as* mutant cells self-renew and differentiate normally, we asked whether the timing of commitment into differentiation is altered. Our results showed a faster and more efficient commitment into differentiation, suggesting that the global levels of H3K9 methylation contribute to irreversibly lock the loss of pluripotency. This observation adds to the notion of H3K9 methylation in general, and SUV39H1 in particular, acting as an epigenetic barrier providing robustness to cell fate changes (9,18,28,42,43). Moreover, our results also underscore the dominance of pluripotency TFs over chromatin modifications (53). We had already shown that NANOG, another key pluripotency TF, controls H3K27me3 levels, particularly during early differentiation (31). Here, we complement this notion with OCT4 controlling H3K9me3 via the *Suv39h1as*/*Suv39h1* tandem. Together, these results place the control of the global levels of heterochromatin marks under the activity of the pluripotency network, extending the concept of the genetic dominance of pluripotency. Whether our observations and conclusions apply to early mouse embryogenesis and to the acquisition and loss of pluripotency *in vivo* is now a question of primary importance. Notably, H3K9 methylation levels are exquisitely regulated during early embryogenesis (54). It is noteworthy that SUV39H1 is absent in oocytes and its expression starts at the 2–4 cell transition stage (35,36), when the reconfiguration of constitutive heterochromatin as chromocenters is initiated. Moreover, the overexpression of SUV39H1 during early embryogenesis leads to developmental defects (13,14): its zygotic overexpression leads to a developmental arrest during compaction. Since *Suv39h1as* displays an exquisite anticorrelated expression to *Suv39h1* upon fertilization (as initially suggested (21) and our data reanalysis has confirmed), whether *Suv39h1as* holds *Suv39h1* expression until the appropriate time to enable the timely establishing of the first heterochromatic structures in the embryo, represents an important question for the future.

## DATA AVAILABILITY

All genome-wide datasets generated for this study are available in GEO (GSE184140): poly-A RNA-seq of EKOiE (GSM5578482 to GSM5578485) and ZHBTC4 cells (GSM5578486 to GSM5578489); total RNA-seq of E14Tg2a (GSM5578490 to GSM5578491); H3K9me3 ChIP-seq (GSM6211351 to GSM6211362); ATAC-seq (GSM6211363 to GSM6211368). Other available datasets in GEO were used: from GSE118898–GSM3350412 to GSM3350417 (44iN), GSM3350424, GSM3350426, GSM3350428 (undifferentiated cells), GSM3350429, GSM3350431, GSM3350433 (differentiating cells); from GSE87822 – GSM2341322 to GSM2341327 (nuclear RNA-seq in ZHBTC4); GSE121708 (single-cell RNA-seq of E4.5 and E7.5 embryos using processed data from: ftp://ftp.ebi.ac.uk/pub/databases/scnmt_gastrulation); GSE45719 (zygote to 16C early embryo single-cell RNA-seq)ṁ—GSM1112490 to GSM1112581, GSM1112603 to GSM1112610, GSM1112656 to GSM1112663, GSM1112694 to GSM1112705, GSM1112766 to GSM1112769. Datasets available from ArrayExpres (EMBL-EBI) were also used: E-MTAB-2958 (RNA-seq of E2.5 to E4.5 mouse embryos).

## Supplementary Material

gkac550_Supplemental_Files

## Notes

Present address: Noa Gil. Friedrich Miescher Institute for Biomedical Research, Basel, Switzerland.

Present address: Nick Owens, Institute of Biomedical and Clinical Science, University of Exeter Medical School, Exeter, UK.
