# Supplementary material for: OCT4 activates a Suv39h1-repressive antisense lncRNA to couple histone H3 Lysine 9 methylation to pluripotency
Source: Nucleic Acids Res. 2022 Jun 28;50(13):7367–79. doi: 10.1093/nar/gkac550 (PMC9303268; doi:10.1093/nar/gkac550)
Supplement: gkac550_Supplemental_Files [file gkac550_supplemental_files.zip › Bernard_etal_SupInfo_revised.pdf]

Supplementary Information

OCT4 activates a *Suv39h1*-repressive antisense lncRNA to couple histone H3 Lysine 9 methylation to pluripotency

Laure D. Bernard et al.

This file contains:

- 8 Supplementary Figures and their legends (pages 2-9)
- Legends for Supplementary Tables (page 10)
- Full description of material and methods (pages 11-19)

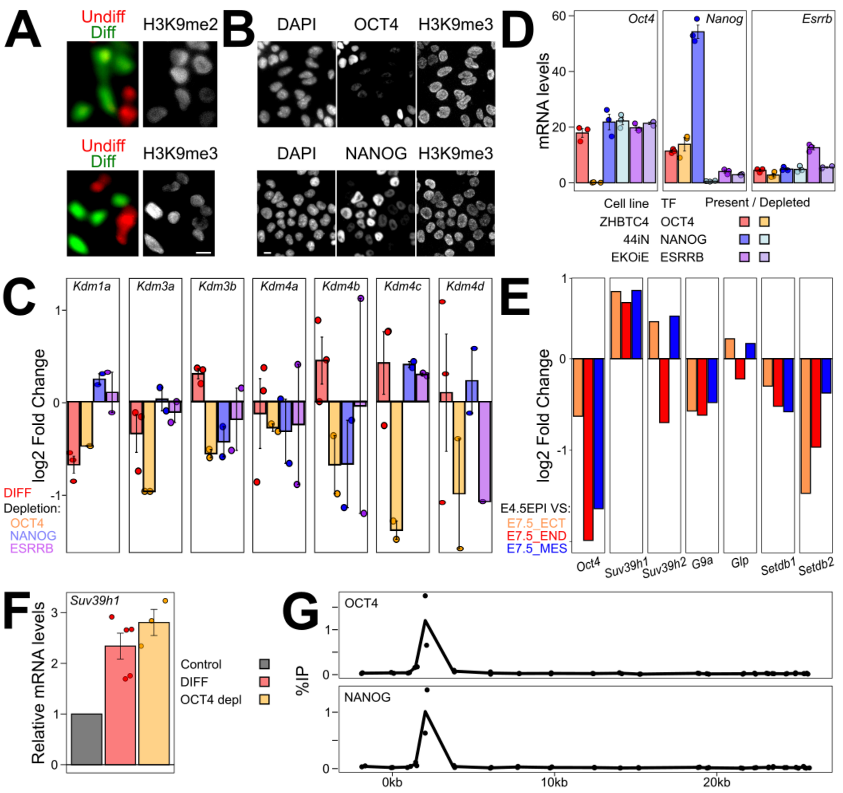

Supplementary figure 1: Additional information for the correlations existing between pluripotency, OCT4 and *Suv39h1*. (A) Illustrative immunofluorescence of H3K9me2 and H3K9me3 in undifferentiated and differentiating ES cells stained and imaged together after independently labelling them with different fluorochromes. **(B)** Illustrative immunofluorescence of OCT4-H3K9me3 or NANOG-H3K9me3 in cultures presenting spontaneously differentiating ES cells. **(C)** Expression of H3K9 de-methylases presented as in Fig.1B **(D)** Expression of *Oct4*, *Nanog* or *Esrrb* upon inducing their depletion in specific dox-inducible knock-out lines. Note in EKOiE the remnant expression of Esrrb produces a truncated, non-functional protein[24]. Each dot represents an independent replicate and the bar the corresponding mean and standard error. **(E)** Log2 fold change of *Oct4* and H3K9 methylases between the three main germ layers of E7.5 embryos and the pluripotent epiblast of E4.5 embryos[27]. **(F)** RT-qPCR validation of *Suv39h1* overexpression upon differentiation (DIFF, 3 days without LIF, red) or upon OCT4 depletion (24h, orange). Each dot represents an independent replicate and the histogram the corresponding mean and standard error. **(G)** ChIP-qPCR validation of OCT4 and NANOG binding at the promoter region of *Suv39h1as* in WT.

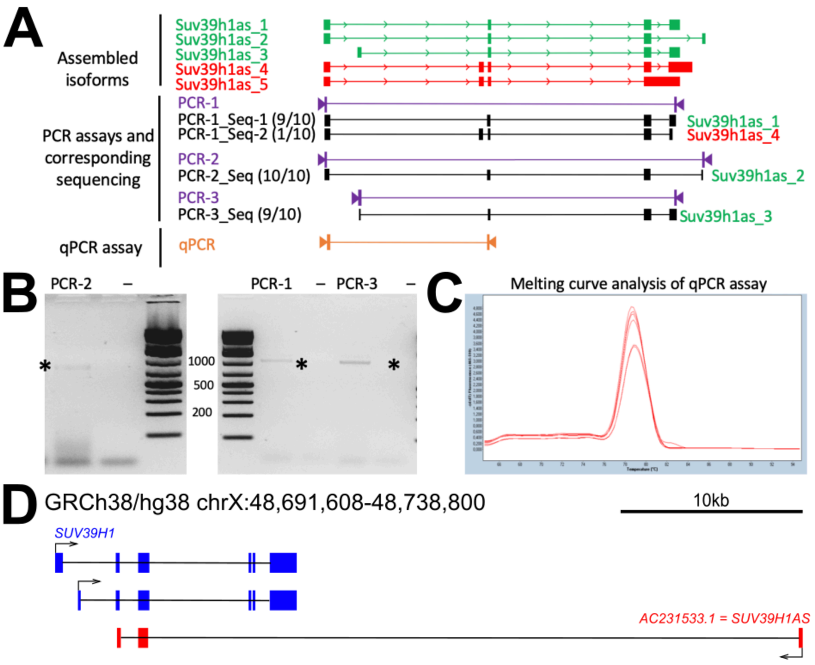

Supplementary figure 2: Additional information on *Suv39h1as* isoforms and conservation. (A-C) *Suv39h1as* isoforms identified in silico using de novo transcript assembly are shown in **(A)** (green and red). Isoforms 1, 2, 3 and 4 were confirmed by cDNA cloning and sequencing, using the primers shown in purple and transforming bacteria with the PCR products shown in **(B)**. However, isoform 4 was found in only 1 out of 10 bacterial colonies, as indicated in (A), and was thus excluded from the final selection. Isoform 5 was never cloned. Moreover, since it has the same 5' end than isoforms 1 and 2, but with an additional small exon, we used melting curve analysis of qPCR reactions **(C)** using primers shown in orange (A), to test whether two different molecular species were amplified. Since this was not the case, isoform 5 was excluded. **(D)** Schematic representation of the *SUV39H1*/ *SUV39H1as* locus in the human genome (Gencode V36 assembly).

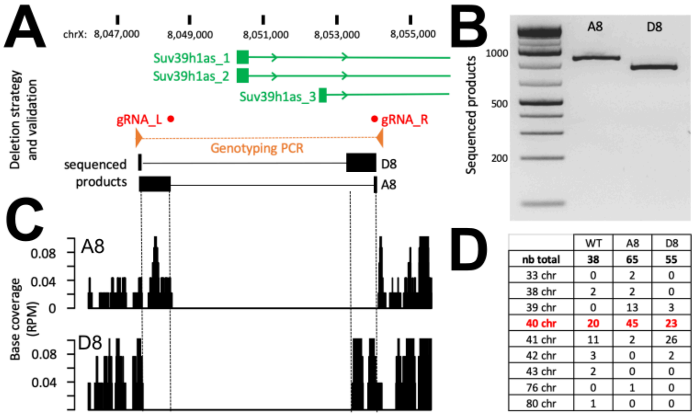

Supplementary Figure 3: Additional information on *Suv39h1as* mutant clones. (A) Schematic representation of the *Suv39h1as* promoter region with the gRNAs used for the deletion and the location of the primers used for validation. **(B)** PCR products using the primers shown in (A) in the two mutant clones. **(C)** Sequencing of sonicated genomic DNA to fully map the deletion boundaries. Overall, these analyses show that the precisely expected deletion was observed for A8 (5.5kb); D8 showed a 5.7kb deletion exhibiting a shift of 700bp compared to A8 but encompassing both *Suv39h1as* promoters. **(D)** Karyotype results for both clones, showing that A8 exhibits a normal karyotype and D8 presents 50% of cells with an extra chromosome.

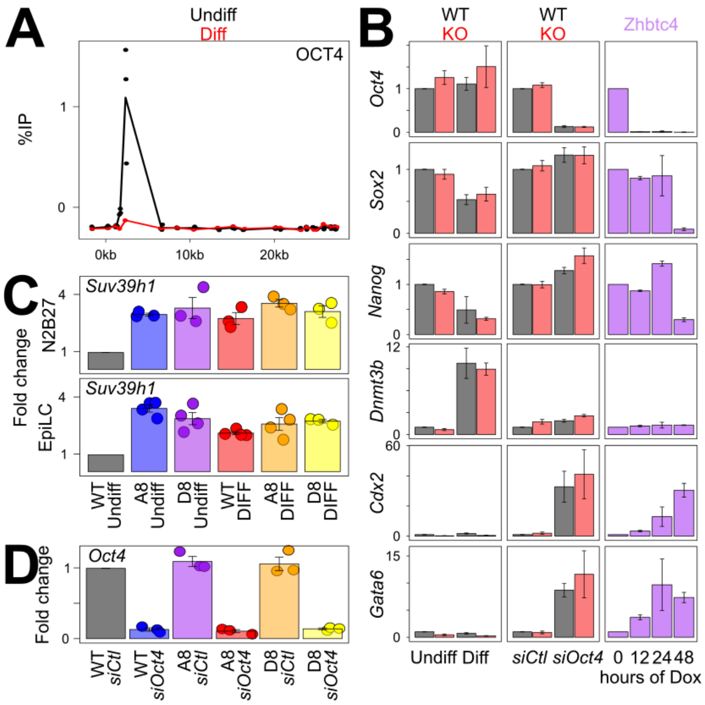

Supplementary Figure 4: Additional information and controls of differentiating ES cells. (A) ChIP-qPCR of OCT4 presented as in Fig.S1 but including a differentiating ES cell sample (3 days without LIF). **(B)** Gene expression marker analysis in the indicated cell lines (top) and conditions (bottom). **(C)** RT-qPCR confirmation of the lack of increased *Suv39h1* upregulation in differentiated *Suv39h1as*-mutant cells obtained by N2B27 or EpiLC assays as compared to WT cells. **(D)** RT-qPCR confirmation of *Oct4* knock-down in WT and *Suv39h1as*-mutant cells.

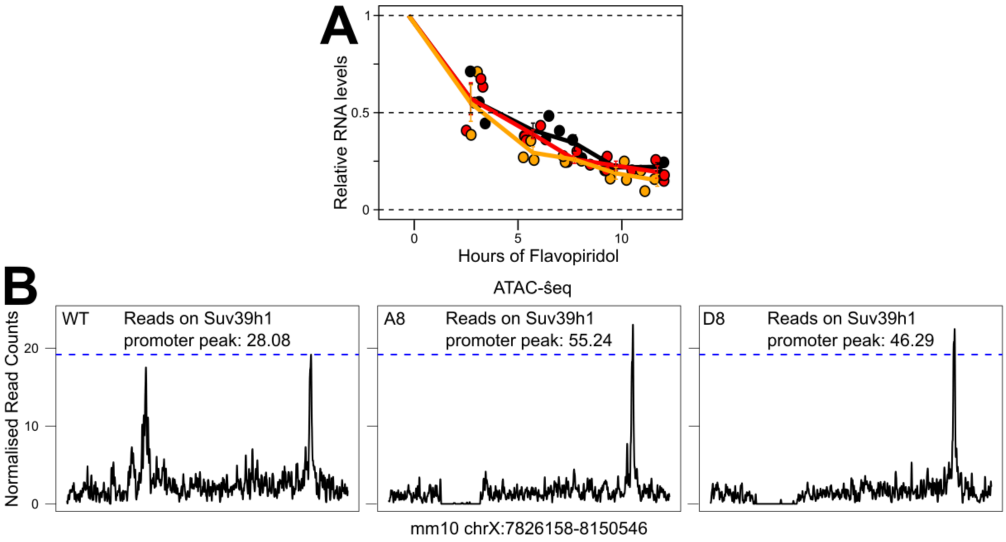

Supplementary Figure 5: Additional support to the transcriptional induction of *Suv39h1* in *Suv39h1as* mutants. (A) Analysis of *Suv39h1* mRNA half-life in WT (black) and *Suv39h1as*-mutant cells (A8, orange; D8, red), performed and presented as in Fig. 2A. **(B)** Accessibility profile across the *Suv39h1*/*Suv39h1as* locus in WT and mutant clones. The blue line denotes the highest height measured in WT cells. The number of normalised reads on the peak identified by MACS2 at the *Suv39h1* promoter are indicated in each panel (Table S2).

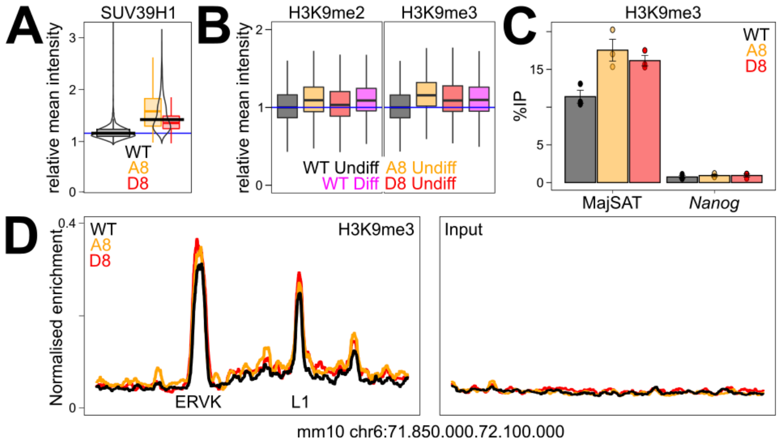

Supplementary Figure 6: Additional data supporting the increase of H3K9 methylation in *Suv39h1as* mutant cells. (A) Violin and box-plots showing immunofluorescence quantification of SUV39H1 mean intensity in WT (black; n= 4048 cells) and mutant cells (A8 – orange; n= 4949 cells and D8 – red; n= 4448 cells). **(B)** Box-plots directly comparing data of Fig.5B with Fig.1A, underscoring the physiological increase of H3K9 methylation in undifferentiated *Suv39h1as* mutant clones. **(C)** ChIP-qPCR of H3K9me3 at Major Satellites and a negative control, the -5kb enhancer of *Nanog*. **(D)** Normalised enrichment of H3K9me3 across the indicated region and corresponding input profiles, in WT and mutant cells. The position of nearly full-length ERVK and L1 elements is indicated.

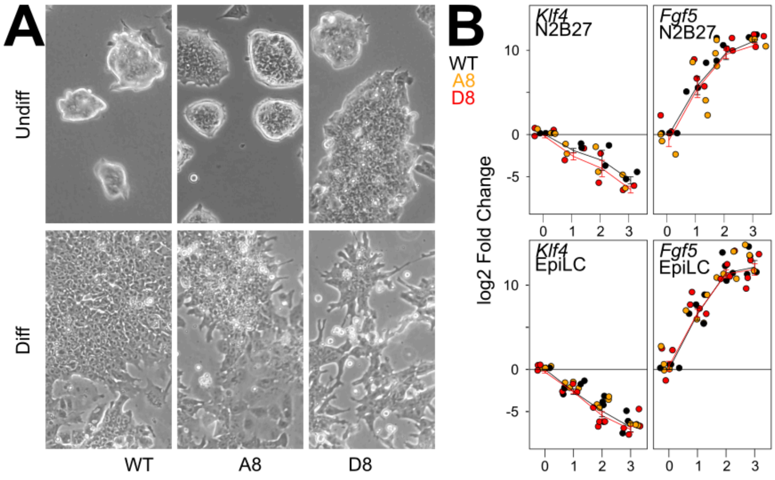

Supplementary Figure 7: Additional information on the differentiation capacity of *Suv39h1as* mutants. (A) Representative photomicrographs of undifferentiated and differentiating WT and mutant cells. **(B)** RT-qPCR analysis of ES (*Klf4*) and differentiation (*Fgf5*) markers during differentiation in N2B27 or in EpiLC conditions, as indicated, in WT and in *Suv39h1as*-mutant cells.

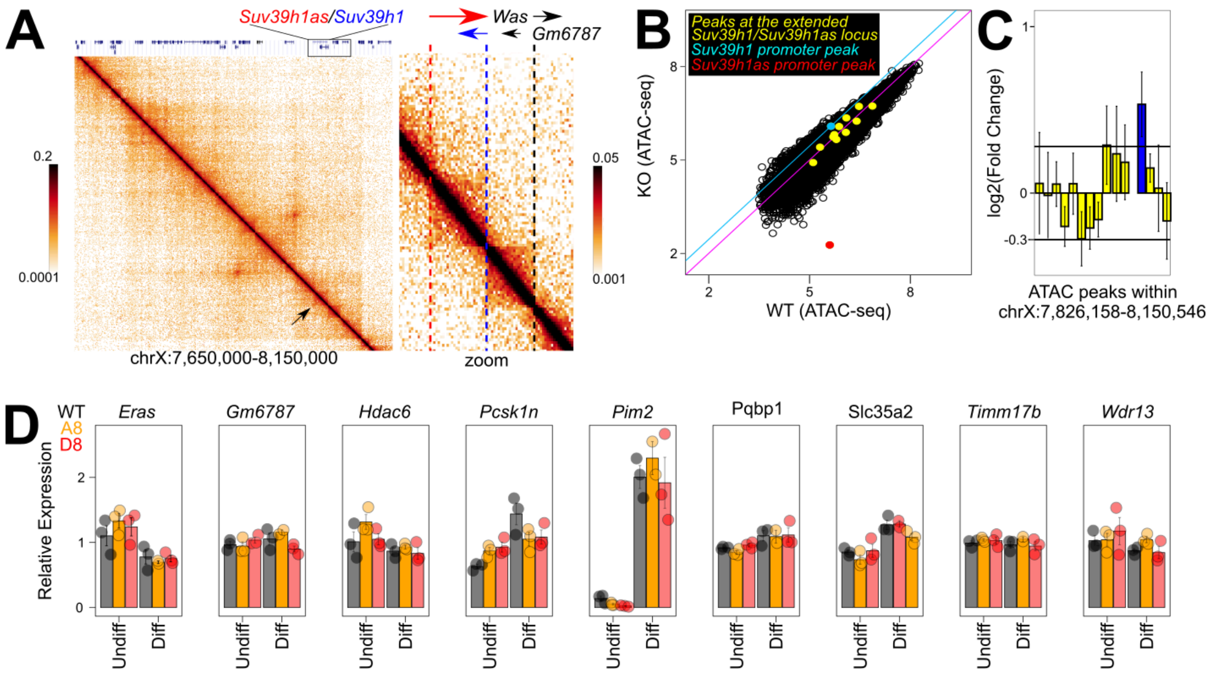

Supplementary Figure 8: Additional data supporting the local effects of *Suv39h1as* deletion. (A) MicroC data visualised on the Higlass.io browser (Hsieh et al. 2019). On the left, a large 500Mb long region; the arrow indicates the position of the *Suv39h1/Suv39h1as* locus. On the right, closer visualisation of the *Suv39h1/Suv39h1as* locus. **(B)** Scatter plot of Drosophila spike normalised accessibility in WT (X-axis) and mutant *Suv39h1as* cells (Y-axis; mean of both A8 and D8 clones), at all identified ATAC-seq peaks in either condition (Table S2). Coloured points correspond to peaks identified in the extended *Suv39h1/Suv39h1as* locus – coordinates shown in (C) – with red corresponding to the *Suv39h1as* promoter region and blue to the *Suv39h1* promoter; the blue diagonal highlights that no other local peak surpasses the increase seen for *Suv39h1* promoter. **(C)** Same data as in (B) but plotted as a log2 fold-change of mutant over WT cells. **(D)** Expression analysis of all genes sharing at least one enhancer.

Description of supplementary tables:

Supplementary Table S1: This table reports gene expression counts of undifferentiated and differentiating ES cells as well as of Dox-inducible depletions of *Oct4*, *Nanog* and *Esrrb*.

Supplementary Table S2: This table reports ATAC-seq data at all peaks identified.

Supplementary Table S3: This table reports H3K9me3 data at all identified enriched regions.

Supplementary Table S4: This table describes H3K9me3 data at all analysed repetitive elements.

Supplementary Table S5: This table describes several reagents (primers, gRNAs, antibodies).

Supplementary material and methods:

Cell lines and generation of A8 and D8 Suv39h1as mutant cells.

Wild-type cells in this study are E14Tg2a ES cells, from which all mutant cells were derived. Dox-inducible knock-out cells as well as OCT-AID cells have been previously described (*Esrrb*: EKOiE[24], *Oct4*: ZHBTC4[25], *Nanog*: 44iN[26], OCT-AID[34]). To generate ES cells deleted for the *Suv39h1as* promoter, several gRNAs were designed using online resources (sam.genome-engineering.org, crispr.mit.edu, chopchop.cbu.uib.no). Highly ranked gRNAs for off-targets as well as on-target predictions score were selected. The gRNAs were cloned into plasmids containing a U6-promoter driven expression cassette along with a puromycin selection cassette or the Cas9 enzyme linked to a mCherry reporter (addgene 51133 and 64324 respectively, see gRNA sequences on Table S5). E14Tg2a cells were lipofected (Lipofectamin 2000; Invitrogen, 11668-019) with 1 µg of the plasmid containing the right deletion site guide and the Cas9 cassette and 3 µg of the plasmid containing the left deletion site guide and a Puromycin cassette. Puromycin (1 µg/ml – Sigma, P9620-10ml) selection was performed for 3 days and mCherry fluorescence checked by microscopy. Resistant cells were plated at clonal density and colonies picked and expanded. Genomic DNA was isolated with NucleoSpin Tissue DNA extraction Kit (Macherey-Nagel, 740952.50), and screened by qPCR and PCR. PCR was performed with LongAmp Taq PCR kit (BioLabs, E5200S) following manufactory's instructions and sequenced. qPCR and PCR primers are available in Table S5. Two knock-out clones, A8 and D8, were selected for further analyses of *Suv39h1* expression. The selected clones were checked by PCR on genomic DNA with primers indicated on Table S5 and with LongAmp Taq PCR kit (BioLabs, E5200S) or Q5 High Fidelity (NEB, M0491S) following manufactory's instructions. The PCR product was validated by agarose gel migration and purified (NucleoSpin Gel and PCR Clean-Up; Macherey-Nagel, 740609.50). PCR products were purified and cloned (ThermoFisher, cat n°450245) as indicated by the manufacturer. Ten bacterial colonies were picked, expanded and checked by PCR and sequencing. All validations are shown in Fig.S3.

Regular cell culture.

Cells were cultured at 37°C, 7% CO2 on 0.1% gelatin-coated plates (SIGMA, G1890-100G) in DMEM+GlutaMax-I (Gibco, 31966-021), 10% FCS (Sigma F7524), 100µM 2-mercaptoethanol (Gibco, 31350-010), 1X MEM non-essential amino acids (Gibco, 1140-035)], supplemented with 10 ng/ml recombinant Leukemia Inhibitory Factor (MILTENYI BIOTEC, 130-099-895). In 2i/LIF medium, cells were grown in N2B27 base [0.5X DMEM/F-12 (Life Technologies, 31331-093), 0.5X Neurobasal (Life Technologies, 21103- 049), 0.5X N2 (Life Technologies, 17502-048), 0.5X B27 (Life Technologies, 17504-044), Insulin 10 µg/ml (Sigma, I-1882), 2mM L-Glutamin (Life Technologies, 25030-024), 37.5 µg/ml BSA (Sigma, A3311-10G) and 0.1mM 2-mercaptoethanol (Life Technologies, 31350010)],

supplemented with 10ng/ml recombinant LIF (MILTENY BIOTEC, 130-099-895), 1µM PD0325901 (Axon 1408) and 3µM CHIRON99021 (Axon 1386). OCT4-AID cells were cultured in 2iLIF. Doxycycline (1000 ng/µL – D3072, Sigma), Puromycin (1 µg/ml – Sigma, P9620-10ml), Flavopiridol (400 nM – Selleckchem, S2679) or Auxin (IAA; 500µM), were extemporaneously added, as indicated. Cells were passaged every 2-3 days, when they reached 70-80% confluency.

Differentiation of ES cells.
Cells cultured in FCS/LIF were differentiated by seeding 300000 cells per well of gelatin-coated 6-wells plate and withdrawing LIF. N2B27 and EpiLC differentiation assays were performed with cells cultured in 2i/LIF for a minimum of 3 passages (9 days). For N2B27 differentiation, 50000 cells per well were seeded on wells of 6-wells plates coated overnight with poly-L-ornithine 0.01% (Sigma, Cat# P4957) at 37°C and 2h with 1X laminin (Sigma, Cat# L2020) and LIF, PD0325901 and CHIRON99021 were withdrawn. For EpiLC differentiation, 230000 cells were seeded per well of 6 wells plates coated with human plasma fibronectin (10 µg/well – F2006, Sigma) and cultured in N2B27 medium containing activin A (20 ng/ml – 338-AC-010, R&D Systems), rhFGF (12 ng/ml – 233-FB, R&Dsystems), and KSR (1% – 10-828-010, Gibco).

Clonal and differentiation commitment assays.
For comparing self-renewal and differentiation capacity of wild-type and *Suv39h1as* clones, 600 cells were plated in gelatin-coated wells of a six well plate. Cells were cultured for 7 days in the indicated media and stained for alkaline phosphatase activity (Sigma, cat. 86R-1KT), following the manufacturer's instructions. Colonies were scored as undifferentiated, mixed and differentiated using a stereo-microscope (NIKON-SMZ1500). For commitment assays, 600 cells obtained every day of differentiation in N2B27 were plated in poly-L-ornithine/laminin-coated wells of a 6 well plate, cultured for 7 days in 2i+LIF and stained for alkaline phosphatase activity.

Assessment of RNA half-lives.
One million cells were plated in a single well of a 6-well plate and treated the next day with Flavopiridol (400 nM – S2679, Selleckchem) for the indicated times. All samples were harvested at the end of the assay for RNA extraction and RT-qPCR analysis.

Oct4 knock-down.
Oct4 knock-downs were performed with siRNAs (Dharmacon, ON-TARGETplus Mouse Pou5f1 siRNA – L-046256-00-0005) and compared to untargeted control siRNAs (On Target plus control pool D-

001810-10-05). Cells were nucleofected with 200 pmol of siRNA using a Mouse ES Cell Nucleofector kit (Lonza, VPH-1001, program A30) and cultured for 24h.

Reverse transcription and real time polymerase chain reaction (RT-qPCR).

Total RNAs were isolated with Trizol (Invitrogen, 15596026) according to the manufacture's protocol and absence of DNA contamination was ensured by additional DNase I digestion (Qiagen, 79254). Reverse transcription (RT) reactions were performed with random hexamers on 0,5 – 2 µg of total RNA according to manufacturer's instructions (First Strand cDNA Kit, Roche, 04379012001). Real-time PCR reactions were performed in duplicate in 384-well plates with a 480 Light Cycler (Roche) using Light Cycler 480 SYBR Green I Master Mix (Roche, 04707516001) and qPCR primers at 0.4µM final concentration. qPCR primers sequences are listed in Table S5. Standard and melting curves were generated to verify the amplification efficiencies (> 85%) and the production of single amplicons. Relative DNA amount was obtained from Cp (Crossing point) calculated from the second derivative of the DNA amplification signal over time. For RT-qPCR experiments, values for gene expression were normalized to the levels of housekeeping gene *Tbp* mRNA. Assays to measure RNA half-lives were normalized to the levels of *28S* mRNA.

RNA-seq analysis and annotation of Suv39h1as.

Poly-A selected RNAs were sequenced by Novogene Ltd (stranded, PE150) and reads aligned to the mm10 genome using STAR[56], quantified by RSEM[57] and counts transformed into Transcripts per million (TPM, Table S1). To annotate *Suv39h1as*, reads were mapped to the genome using Hisat2[58]. Bam files were used to build new transcript models with Stringtie[59] based on the Gencode (vM12) gtf (default parameters except -m 300). The resulting gtf files were merged (default parameters, except -m 300 -c 0.5 -F 0.5 -f 0.05), and all *Suv39h1as* isoforms annotated. Only those experimentally validated by Topo-cloning of poly-A selected cDNAs and RT-qPCR quantifications were retained (Fig.S2)

Chromatin immunoprecipitation (ChIP).

Ten million cells were crosslinked either for 45 min with DSG 1X plus 10 min in Formaldehyde (FA) 1% in PBS 1X for TF binding analysis or only for 10 min in FA 1% for histone modification analysis. Formaldehyde was quenched with 125 mM glycine for 5 min at room temperature. Nuclei were prepared in 1ml of ice-cold swelling buffer (25 mM Hepes pH7.95, 10 mM KCl, 10 mM EDTA) freshly supplemented with 1× protease inhibitor cocktail (PIC-Roche, Cat# 04 693 116 001) and 0.5% IGEPAL (Sigma, Cat#I8896) for 20 min on ice and 50 passes in a dounce homogenizer. Nuclei were then resuspended in ice-cold D3 buffer (0.1% SDS, 15 mM Tris pH 7.6, 1 mM EDTA), freshly supplemented

with 1×PIC, and sonicated using a Covaris M220-Setpoint at 6°C and 10 (FA only) to 15 (DSG/FA) cycles with the following parameters for each cycle: 60 sec duration, peak power of 67W, duty factor of 15% and cycles/burst of 500. A delay of 45 sec is added at the end of each cycle. Result of the average power is 10W. For ChIP-seq of H3K9me3, sonication was performed with a Bioruptor Pico (Diagenode) for 7 cycles divided into 30 s ON–30 s OFF sub-cycles at maximum power, in circulating ice-cold water. After centrifugation (15 min, 14000 rpm, 4 °C), the supernatant was stored at −80 °C until use. 20 µl were used to quantify the chromatin concentration and check DNA size (typically 200-600 bp for ChIP-qPCR and 150-350 bp for ChIP-seq). Fifteen to 20 µg of chromatin were used for each ChIP-qPCR after pre-clearing it for 1.5 hours rotating on-wheel at 4 °C in 1 ml of TSE150 (0.1% SDS, 1% Triton X-100, 2 mM EDTA, 20 mM Tris-HCl pH8, 150 mM NaCl) buffer containing 50 µl of protein G Sepharose beads (Active Motif, Cat#37499) 50% slurry, previously blocked with BSA (0.5 mg/ml; Roche, Cat# 10711454001) and yeast tRNA (1 µg/ml; Roche Cat# 10109495001). For ChIP-seq experiments, 20 µg mouse ES cell chromatin was combined with 1.6 µg (1:25 ratio) Drosophila S2 cells spike chromatin. Immunoprecipitations were performed overnight rotating on-wheel at 4 °C in 500 µl of TSE150. 20 µl were set apart for input DNA extraction and precipitation. 50µl of blocked protein G beads 50% slurry was added for 2h rotating on-wheel at 4 °C. Beads were pelleted and washed for 5 min rotating on-wheel at room temperature with 1 ml of buffer in the following order: 2× TSE150, 1 × TSE500 (as TSE150 but 500 mM NaCl), 1× washing buffer (10 mM Tris-HCl pH8, 0.25M LiCl, 0.5% IGEPAL, 0.5% Na-deoxycholate, 1 mM EDTA), and 2 × TE (10 mM Tris-HCl pH8, 1 mM EDTA). Elution was performed in 100 µl of elution buffer (1% SDS, 10 mM EDTA, 50 mM Tris-HCl pH 8) for 15 min at 65 °C after vigorous vortexing. Eluates were collected after centrifugation and beads rinsed in 150 µl of TE-1%SDS. After centrifugation, the supernatant was pooled with the corresponding first eluate. For both immunoprecipitated and input chromatin, the crosslinking was reversed overnight at 65 °C, followed by proteinase K treatment, phenol/chloroform extraction and ethanol precipitation. For ChIP-qPCR, input and IP samples were analysed using primers in Table S5. The 2dCt method was used. All values were corrected to the input. The antibodies used and their working dilution are indicated in Table S5.

ChIP-seq library and sequencing.

End repair: Precipitated DNA was resuspended in 37.5µl of water and mixed with 2µl of 10mM dNTPs, 5µl of NEB T4 ligase buffer, 2.5µl of NEB T4 polymerase (Cat# M0203L), 0.5µl of NEB Klenow polymerase (Cat# M0210L) and 2.5µl of NEB T4 PNK (Cat# M0201L). Samples were incubated 30 min at 20°C in a thermocycler. DNA was purified with SPRI beads: 90µl of SPRI bead suspension and 50µl isopropanol were added and samples transferred to a 96 well plate. After incubating for 5 min, the plate was put on a 96S Super Ring Magnet (Alpaqua, Cat# A001322), beads were allowed to separate completely, and the supernatant removed without disrupting the bead pellet. Beads were washed

twice with 200µl of 70% Ethanol and the supernatant completely removed. DNA was eluted in 21µl of water.

A-Tailing: 20µl of sample were mixed with 2.5µl of NEB Buffer #2, 1µl of 5mM dATP, 1.5µl of NEB Klenow 3'-5' exo minus (Cat# M0212L), and incubated at 37°C for 30min in a thermocycler. DNA was purified with SPRI beads as before, but using a volume of 45µl of beads and 25µl isopropanol. DNA was eluted in 20µl of water.

Adaptor ligation: 19.5µl of sample were mixed with 2.5µl of NEB T4 ligase buffer, 1µl of a 1µM solution of annealed adapters, and 2.5µl of NEB concentrated T4 ligase (Cat# M0202M) and incubated overnight at 16°C. DNA was purified with SPRI beads as before, but using a volume of 35µl of beads and no isopropanol, eluting in 20 µl of water. Adaptors were designed in house. 22.5µl each of 40µM ssDNA Barcoded and Universal adaptor solutions were mixed with 5µl of NEB buffer #2, and annealed in a thermocycler. Barcoded adaptor: 5'P-GATCGGAAGAGCACACGTCTGAACTCCAGTCAC-IIIIII-NNNNNNNN-ATCTCGTATGCCGTCTTCTGCTTG. 5'P indicates the presence of a 5'phosphate group. IIIIII represent the six base index nucleotides, and NNNNNNNN a stretch of 8 random nucleotides used for single molecule barcoding.

Universal adapter: AATGATACGGCGACCACCGAGATCTACACTCTTTCCCTACACGACGCTCTTCCGATC*T. * indicates the presence of a phosphorothioate bond between the last C and T.

Library amplification: 19.5 µl of sample were mixed with 1µl of a 1:10 dilution of Quant-iT Picogreen dye (Invitrogen, Cat# P11496), 25µl of KAPA HiFi HotStart 2x master Mix (Kapa Bioscience Cat# KK2502), 1µl of 10µM PCR 1.0 and 1µl of each primer. The amplification mix was distributed in two wells of a LightCycler 384 plate (Roche, Cat# 4729749001) and on a LightCycler 480 II instrument (Roche, Cat# 05015243001) using the following program: 1' at 98°C; N cycles: 10" at 98°C; 20" at 64°C; 45" at 72°C. The number of cycles N was determined on real time by monitoring the fluorescence such that the amplification was stopped during the exponential phase. Samples were removed from the plate and purified with SPRI beads using 54µl beads, no isopropanol, and eluting in 40µl of water. 1µl was used to measure the DNA concentration with a Qubit 3 and the provided reagents (Invitrogen, Cat#Q33218). 1µl of DNA was used to check fragment size with a D1000 High Sensitivity Screentape and appropriate reagents (Agilent, Cat# 5067-5584, Cat# 5067-5585) on an Agilent 2200 Tapestation. ChIP-seq was sequenced 1x75bp on a NextSeq 500 with NextSeq 500/550 High Output Kit v2.5 (75 Cycles; Illumina, Cat# 20024906).

ATAC-seq and sequencing.

Chromatin accessibility was analyzed using an adaptation of the ATAC-seq (transposase accessible-chromatin-seq) protocol previously described[28]. Briefly, 100,000 viable mouse ES cells were harvested, washed with PBS and mixed with 5000 Drosophila S2 cells. Instead of using lysis buffer to isolate nuclei,

cells were pelleted by centrifugation for 5 min at 500g at 4°C, resuspended in 50 µl of transposition reaction mix (25 µl of Tagmentation DNA buffer, 2.5 µl Tagment DNA enzyme (Illumina Tagment DNA TDE1 Enzyme and Buffer Kits, Cat# 20034197) and 22.5 µl nuclease-free H2O) and incubated for 30 min at 37 °C with gentle agitation. Reactions were stopped by adding the appropriate volume of Binding Buffer (Qiagen MinElute PCR Kit) and the DNA was purified using the Qiagen MinElute PCR Kit according to manufacturer's protocol. The purified DNA, eluted in 10 µl, was either stored at -20°C or used directly for library preparation. ATAC-seq libraries were generated using 10 µl transposed DNA, custom made Illumina barcodes previously described (Buenrostro et al., 2013) and KAPA HiFi HotStart (KapaBiosystems KM2602) for PCR amplification. The number of PCR cycles for PCR amplification was determined using qPCR. Following PCR-amplification, libraries were purified using SPRI beads, using a sample to bead ratio of 1: 1.4. One microliter of the library was used to measure the concentration with the Qubit and the provided reagents (Invitrogen, Cat# Q33218) and to determine fragment size with a D1000 High Sensitivity Screentape and appropriate reagents (Agilent, Cat# 5067-5584, Cat# 5067-5585) on an Agilent 2200 Tapestation. ATAC-seq libraries were paired-end sequenced on Illumina NextSeq500 using 65 bp paired-end reads.

Bioinformatic processing of ChIP-seq and ATAC-seq.

For Chip-seq, reads were aligned with Bowtie 2 to the mm10 and dm6 genome, with options "--local --very-sensitive-local --dovetail"[60]. For reads with multiple alignments, only one was randomly kept. All libraries were constructed with custom unique molecular identifiers. Therefore, reads aligning with identical position, strand and barcode were treated as duplicates and collapsed using picard and the tool UmiAwareMarkDuplicatesWithMateCigar; UMIs were added to the bam files using fgbio AnnotateBamWithUmis tool. To calculate normalisation factors for ChIP and input fractions, all reads were filtered for those with a single discovered alignment and calculated as dm6 reads / total number of reads. ChIP normalisations factors were additionally corrected to those calculated in the input. These normalisation factors were then applied to correct coverage and read per million counts, calculated without filtering out multimappers such that repetitive elements can be quantified. Global analysis of repetitive elements was performed using RepEnrich tool[53]. RepEnrich estimated read counts per sample were rounded for use with DESeq2[55] using drosophila spike-in normalization factors as scaling factors. To identify regions enriched for H3Kçme3, we used a strategy previously described[40], where peaks were called against relevant inputs for all samples. Enriched genomic domains were called using a 2-kb sliding window algorithm, with a sliding step of 1-kb. For every 2-kb window in the genome, the number of reads falling in that window for a given sample (normalized to the number of reads sequenced) was divided by the number of reads in that window for the corresponding input file (also normalized to the number of reads sequenced). Divide-by-zero errors were avoided by spiking in

a small value into both numerator and denominator (0.25 reads per million reads sequenced). Enriched domains were initially formed by taking all 2-kb windows whose signal-over-input value exceeds 80% percentile of the signal-over-input values distribution threshold.

For ATAC-seq, Nextera adapters were trimmed using cutadapt tool. Reads were aligned with Bowtie 2 to the mm10 and dm6 genomes, with options "-I 0 -X 1000 --no-discordant –no mixed --dovetail". Reads were additionally deduplicated using samtools and filtered for those with a single discovered alignment. Peaks were called using MACS2 with "callpeak -q 0.1 -g mm --keep-dup all"[61]. Peaks were further filtered to those identified in either 2 WT replicates out of 2 or in 3 mutant replicates out of 4 (2 in A8 and 2 in D8).

RNA/DNA Fluorescent In Situ Hybridization (FISH).

Using the online tool "Stellaris RNA FISH probes designer" from LGB biosearch laboratories, single-strand probes for *Suv39h1* (47 oligos, 30 in exons, see sequences Table S5) and *Suv39h1as* (35 exonic oligos, see sequences Table S5) were designed for single-molecule FISH (smFISH). DNA probes for DNA-FISH were generated by nick translation (Vysis Nick Translation Kit; Abbott, cat. 32-801300) using a fosmid clone (WIBR1-2188H11 – from Children's Hospital Oakland Research Institute, bacpac.chori.org) covering the entire locus. Cells were fixed in Formaldehyde 4%, quenched with Glycine (1M) and cytospun (Cytospin3, Shandon, at 400 rpm for 5 min with a low acceleration) onto slides that were kept in Ethanol 70% at 4°C until use. Slides were washed in 100% ethanol for 2 min and air dried. For each spot, 10 µL of hybridization cocktail (SSC2X – S6639-1L, Sigma; Dextran – Life technologies, 5%; Formamide 10% – Sigma F9037; 2 µg/µL E. Coli tRNAs – Sigma 10109541001; 5 mmol/L Ribonucleoside Vanadyl Complex – NEB S1402S; 0,5 µg/µL BSA – NEB B9001S) and smFISH probes for *Suv39h1* and *Suv39h1as* (each at 0,6 µmol/L) were used for overnight incubation into a humid chamber at 37°C. Slides were washed in fresh SSC 2X/Formamide 10% for 30 min at 37°C, mounted and counterstained with Vectashield Antifade Mounting medium with DAPI (Vector Laboratories, H-1200-10). Sm-FISH images were acquired with an inverted Nikon Eclipse X microscope equipped with: X63 oil immersion objective (N.A1.4); LUMENCOR excitation diodes; Hamamatsu ORCA-Flash 4.0LT camera; NIS Elements 4.3 software. The position of each image was recorded on the microscope. Subsequently, the coverslips were removed and the slides washed 3 times in washing medium (4X SSC, 0,2% Tween-20) at 37°C, and treated with RNaseA 10U/ml (Invitrogen, cat. EN0531) in 2XSSC at 37°C for 1h. DNA denaturation was performed in 50% formamide/2XSSC at 80°C for 30min. Slides were dehydrated in cold ethanol and hybridized overnight in a 50% Formamide/ 2X Hybridization cocktail (4X SSC – Sigma, S6639-1L; 20% Dextran sulfate – Life technologies; 2 mg/mL Bovine Serum Albumine –NEB B9001S; and 40 mM Ribonucleoside Vanadyl Complex– NEB S1402S) at 37°C with 0.3 ng of DNA-Fish probe, 3ul of mouse Cot1 DNA (Invitrogen, cat 18440016) and salmon

sperm DNA (Invitrogen, cat.15632011), previously denatured in Formamide (7' at 75°C). After overnight hybridization of the probes, the slides were washed 3 times in 50% Formamide/2X SSC buffer at 37 °C for 5 min and 3 times in 2XSSC buffer at 37 °C for 5 min, mounted with Vectashield containing DAPI and imaging on the previous recorded positions.

Western-blot.

Cell were lysed in in Laemmli buffer (1 000 000 cells per µL ; #1610747, BioRad) at 95°C for 5 min and samples run in a mini-PROTEAN® TGX Stain-Free Precast Gel (Bio-Rad, 456-8086) in 25 mM Tris, 0,21 M Glycine, 50% SDS at 120V with constant voltage and transferred onto nitrocellulose membranes (Life Science, 10600003) in a 25mM Tris, 0.21 M glycine, 20% ethanol solution. The membrane was blocked in phosphate-buffered saline (PBS; 0.8 g/L NaCl, 0,02 g/L KCl, 0.144 g/L Na2HPO4, 0.024 g/L KH2PO4, pH = 7.2) with 0.1% Tween (PBST), 5% Bovin Serum Albumin (BSA) for 1 h and incubated in 3 ml of PBST 5% BSA with different antibodies listed Table S5, overnight at 4°C. Membranes were washed 3 times for 5 min in PBST and incubated with Pierce® goat anti-rabbit IgG-HRP conjugated secondary antibody (Thermo Scientific #314666, 0,1 µg/ml or 50 ng/ml). Membranes were washed in PBST and developed using a Pierce® ECL Western Blotting substrate kit (Thermo Scientific, #32109) or Pierce® ECL plus Western Blotting substrate kit (Thermo Scientific, #32134) for 5 min or 1 min at RT and luminescence detected using a ChemiDoc MP Imaging Systems with Image LabTMTouch Software Version 2.2.0.08.

Immunostainings.

Cells were trypsinized, counted and resuspended at 1 million/ml in FCS free medium (DMEM-Glutamax/100 mM 2-mercaptoethanol/NEAA 1X) into sterile 1.5 ml Eppendorf tubes. To ensure direct comparison of wild-type and mutant cells, they were then individually incubated either with 10 µM Rhodamine Red dye (InVitrogen, Cat#CMTPX C34552) or 1µM Deep Red dye (InVitrogen, Cat#C34565) for 20 to 40 min at 37°C. The labeled cells were then collected by centrifugation, washed with PBS1X, resuspended in DMEM/10%FCS+LIF medium and mixed at a 1:1 ratio for Rhodamine and Deep Red labelled cells (usually ~0.4M each). 800 000 mixed cells were seeded onto Poly-L-Ornithine/Laminin coated single wells of a µ-slide 4 well Ph+ibiTreat (Ibidi GmbH Ref#80446) and incubated for 6H at 37°C and 7% CO2. Cells were then fixed directly into the well with freshly prepared PFA 4% (Fisher Scientific, Cat#16431755) for 10 min at room temperature in the dark and washed twice in PBS1X for 10 min. Cells were permeabilized with PBS1X/0.1% Triton X-100 (Sigma, Cat#T8787) for 10 min at room temperature. After three washes with PBS 1X, cells were blocked with PBS 1X/3% Donkey Serum (Sigma, Cat#D9663) for 30 min in the dark and incubated overnight with primary antibodies (diluted in PBS 1X/10% DS). Following three washes with PBS, 1h incubation with secondary antibodies at room

temperature in the dark and 3 washes with PBS 1X, nuclei were counterstained with DAPI (Sigma, Cat#D9542), washed in PBS1X and imaged with an inverted Nikon Eclipse X microscope equipped with: X20/0.45 (WD 8.2-6.9) objective; LUMENCOR excitation diodes; Hamamatsu ORCA-Flash 4.0LT camera; NIS Elements 4.3 software. Quantifications were performed using Cell Profiler[62]. For each experiment, Rhodamine/Deep Red labelled cells were attributed using the FlowJo software. For each experiment, the fluorescence intensity of mutant cells was normalised to the median of the corresponding wild-type cells intensities imaged on the same spot.

Additional references

56. Dobin, A. et al. STAR: ultrafast universal RNA-seq aligner. Bioinformatics 29, 15–21 (2013).

57. Li, B. et al. RSEM: accurate transcript quantification from RNA-Seq data with or without a reference genome.
BMC Bioinformatics 12, 323 (2011).

58. Kim, D., et al. Graph-based genome alignment and genotyping with HISAT2 and HISAT-genotype.
Nat Biotechnol 37, 907–915 (2019).

59. Pertea, M. et al. StringTie enables improved reconstruction of a transcriptome from RNA-seq reads.
Nat Biotechnol 33, 290–295 (2015).

60. Langmead, B., and Salzberg, S.L. Fast gapped-read alignment with Bowtie 2. Nat Methods 9, 357-359 (2012).

61. Feng, J., et al. Identifying ChIP-seq enrichment using MACS.
Nat Protoc 7, 1728-1740 (2012).

62. Carpenter, A. E. et al. CellProfiler: image analysis software for identifying and quantifying cell phenotypes.
Genome Biol 7, R100 (2006).
